# Identification and bioinformatics analysis of cilia-associated gene families in *Euplotes amieti* (Ciliophora, Hypotrichia)

**DOI:** 10.3389/fmicb.2025.1486189

**Published:** 2025-05-13

**Authors:** Liheng Shen, Xiaobing Xiong, Zixiang Xu, Yingli Liu, Yan Sheng, Xin Sheng

**Affiliations:** ^1^Department of Biochemistry, Zunyi Medical University, Zunyi, China; ^2^Laboratory of Basic Medical Morphology, Zunyi Medical University, Zunyi, China

**Keywords:** *Euplotes amieti*, next-generation sequencing, cilia-associated genes, ciliopathy, bioinformatics analysis

## Abstract

**Introduction:**

Ciliates serve as pivotal model organisms for investigating the protein composition and regulatory mechanisms underlying cellular processes. This study systematically explores the structural and functional characteristics of cilia-associated genes in *Euplotes amieti*, aiming to elucidate their roles in ciliogenesis and cilia-related pathways.

**Methods:**

The macronuclear genome of *E. amieti* was sequenced using next-generation sequencing (NGS). Cilia-associated genes were identified via BLASTP analysis against homologs in hypotrich ciliates (*Euplotes octocarinatus*, *Stylonychia lemnae*, and *Oxytricha trifallax*). Functional annotations, including Non-Redundant (NR) classification, Pfam domain prediction, and Gene Ontology (GO) enrichment, were performed. Pathway enrichment analysis identified associated metabolic and signaling pathways. Experimental validation included quantitative polymerase chain reaction (QPCR) of cilia-related gene families, RNA interference (RNAi) targeting ARL2BP and DYNLRB2, and immunofluorescence staining to assess microtubule arrangement.

**Results:**

A total of 418 cilia-associated genes were identified, with 44 conserved across the four hypotrich ciliate species. Functional categorization revealed kinases, dyneins, tubulins, and autophagy-related proteins. Pfam annotations predicted three conserved domains. GO terms were enriched in tubulin binding, cilia assembly, and microtubule-based movement. Pathway analysis implicated these genes in adenine ribonucleotide biosynthesis, glycolysis/gluconeogenesis, Wnt, and AMP-activated protein kinase (AMPK) signaling. QPCR showed significant downregulation of cilia-related proteins during mitosis. RNAi of ARL2BP and DYNLRB2 increased mortality, reduced motility, and disrupted cortical microtubule organization via immunofluorescence. Thirty-nine hub genes were strongly linked to ciliopathies.

**Discussion:**

Cilia-associated genes in *E. amieti* are integral to DNA replication, energy metabolism, intercellular communication, and morphogenesis. The conserved hub genes associated with ciliopathies suggest evolutionary preservation of ciliogenesis regulation. ARL2BP and DYNLRB2 are functional important in ciliary dynamics and structural integrity. This study provides crucial insights into the roles of cilia-associated genes in ciliates, advancing understanding of ciliogenesis mechanisms and their implications for ciliopathy research.

## Introduction

Cilia are evolutionarily conserved organelles that project from the cell surface in a wide range of species, from vertebrates to ancient protozoa. They feature a highly conserved ultrastructure, comprising a basal body derived from a mother centriole, a microtubule-based axoneme, and a distinct ciliary membrane (Fliegauf et al., 2007; Klena and Pigino, 2022). Given their ubiquitous presence, cilia are crucial for numerous physiological processes, broadly classified into motility and signal transduction (Marshall and Nonaka, 2006; Chen et al., 2006). Motile cilia facilitate the movement of cells or extracellular fluids, while non-motile cilia play a role in sensing environmental signals (Fliegauf et al., 2007; Mitchison and Valente, 2017). Various signaling pathway receptors, including Sonic Hedgehog (SHH), platelet-derived growth factor receptor (PDGFR), Wnt, Hippo and mechanistic target of rapamycin (mTOR) are localized on the ciliary membrane (Wheway et al., 2018; Wallmeier et al., 2020). In addition, motile cilia in ciliates are also important for sensing environmental signals. These organelles are critical to how ciliates interact and perceive their environment. Consequently, cilia dysfunction can lead to a spectrum of ciliopathies, such as nephronophthisis, neurodevelopmental disorders, subfertility, primary ciliary dyskinesia, and retinal or skeletal disorders (Wallmeier et al., 2020; Hildebrandt et al., 2009; Karalis et al., 2022; Handa et al., 2020; Chen et al., 2021). These ciliopathies are often attributed to mutations in single cilia-associated genes (Lee and Gleeson, 2011; Shaheen et al., 2016). Therefore, identifying these genes and elucidating their roles in cilia motility, signal transduction, and ciliogenesis is critical for understanding cilia function and related disorders.

Although it is established that cilia are composed of hundreds of proteins, many of which are conserved across eukaryotes (Pazour et al., 2005; Ishikawa et al., 2012; Narita et al., 2012), the precise functions of these cilia-associated proteins remain largely unexplored. *Euplotes*, a complex hypotrich ciliate with global distribution and high adaptability to marine, freshwater and terrestrial environments, serves as an ideal model for studying microtubule assembly, ciliary structure and function, as well as ciliogenesis and ciliary gene localization (Fleury, 1991; Gu and Zhang, 1991; Sheng et al., 2011a; Pucciarelli et al., 2022). The detailed morphology of the cortical cytoskeleton in *Euplotes eurystomus* was first described by Wise in 1964 (Wise, 1965), with subsequent studies by Fleury in 1991 further elucidating the dynamics of the cytoskeleton during morphogenesis (Fleury, 1991). Immunofluorescence labeling has recently revealed distinct variations in the cortical tubulin composition surrounding the ciliary basal body (Kloetzel and Brann, 2012; Lin et al., 2013; Sheng et al., 2017). These findings highlight the diverse and intricate nature of the protein composition within the basal body-related cortex. However, the protein composition and function of cilia-related cortical microtubule skeletons in *Euplotes* remain unknown.

Occasional structure and functional studies have been conducted on some common cilia-associated proteins, such as γ/ε-tubulin (Sheng et al., 2017; Garreau de Loubresse et al., 2001; Marziale et al., 2008; Sheng et al., 2011b; Ross et al., 2013; Joachimiak et al., 2018), centrin (Zhao et al., 2011, Zhao et al., 2018), Bardet-Biedl Syndrome (BBS) proteins (Valentine et al., 2012; Price et al., 2012; Seixas et al., 2010), and the centriole proteome POC1 (Meehl et al., 2016). The extensive use of omics technologies has facilitated a systematic and comprehensive analysis of cilia-associated proteins in ciliates. For instance, 24 previously unidentified basal body proteins were discovered in *Tetrahymena thermophila* (Kilburn et al., 2007), 147 novel proteins in the ciliary membrane of *Paramecium tetraurelia* (Yano et al., 2013), and 115 transition zone proteins from *Chlamydomonas* (Diener et al., 2015). Recently, Chen identified 130 encystment-related proteins in *Euplotes encysticus* using 2-D electrophoresis and MS, enhancing our understanding of the morphology and molecular mechanisms in *Euplotes* encystment (Chen et al., 2018). Thus, the screening of cilia-associated proteins through omics approaches provides foundational data for subsequent functional investigations. The identification of ciliary and ciliopathy genes in *Caenorhabditis elegans* dates back to 2006 (Chen et al., 2006), and there is a growing body of research suggesting protozoa as suitable model organisms for ciliopathy research (Soares et al., 2020; Valentine and Van Houten, 2021). Nevertheless, research on cilia-associated genes or proteins in *Euplotes* remains limited.

This study involved the screening of 418 cilia-associated proteins through the sequencing of the macronuclear genome and transcriptome of *Euplotes amieti*. The selection of NGS for genome sequencing was based on its ability to provide comprehensive genetic data, enabling the identification of cilia-associated genes with high precision. The use of bioinformatics tools such as GO and KEGG was driven by their capacity to categorize and annotate these genes, offering insights into their potential roles in ciliary assembly and function. For functional validation, RNA interference (RNAi) was chosen to target specific genes, such as ARL2BP and DYNLRB2, based on their known involvement in ciliary structure and motility. This approach allowed us to confirm the functional relevance of the identified genes and their potential role in ciliopathies. Comprehensive structural analyses of these gene families were conducted, including Gene Ontology (GO) annotations, Kyoto Encyclopedia of Genes and Genomes (KEGG) pathway enrichment, and protein-protein interaction (PPI) network analyses. Additionally, the structural characteristics of these genes and their potential associations with ciliopathies were investigated. This research aims to provide valuable insights into the regulation of cilia-associated proteins in *Euplotes*, particularly in relation to cortical organization and cilia assembly.

## Materials and methods

### Collection and preparation of *E. amieti*

*E. amieti* specimens were collected from a farmland pond in Qingpu District, Shanghai. The DNA extraction kit used was sourced from TaKaRa, while genome sequencing and annotation were conducted by Beijing Nuohe Zhiyuan Technology Co., Ltd. Transcriptome sequencing was carried out by Shanghai Pacino Biotechnology Co., Ltd.

To cultivate and collect the hypotrich ciliate *E. amieti*, the organisms were fed with *Chlorogoium elongatum*. After feeding, the *E. amieti* were starved for 5 days prior to DNA or RNA extraction. The starved cells were filtered using three layers of gauze to eliminate larger impurities. Subsequently, the cells were concentrated on qualitative filter paper to remove smaller impurities. The cells were then resuspended in pure water and centrifuged at 4,000 r/min for 5 min, preparing them for further use.

### DNA extraction and genome sequencing of *E. amieti*

DNA extraction followed the protocol of the TaKaRa DNA Extraction Kit. Agarose gel electrophoresis was utilized to assess the purity and integrity of the DNA, while the purity (*A*_260_/*A*_280_ ratio) was measured using a Nanodrop spectrophotometer. Precise DNA concentrations were quantified with a Qubit fluorometer. Qualified DNA samples were fragmented into 350 bp lengths using Covaris technology. The DNA library was constructed using the NEB Next^®^ Ultra DNA Library Prep Kit (NEB, USA) and sequenced with the Illumina NovaSeq PE150 platform. Genome assembly was performed using the “careful” mode in SPAdes, followed by sequence fusion using CAP3. Sequences shorter than 500 bp were aligned to those longer than 500 bp using BLAST to filter out low-quality reads. This process removed sequences with a percentage of consistency ≥ 90% and coverage ≥ 80%, as well as bacterial, archaeal DNA sequences, non-telomeric sequences, mitochondrial genome sequences, and 11 sequences shorter than 100 bp. A total of 9,508 reads were excluded. The clean contigs were used for whole genome annotation.

### RNA extraction and transcriptome sequencing

RNA was extracted using the TaKaRa RNA extraction kit and fragmented into 300 bp fragments via ion interruption. A 450 bp library was then constructed and subjected to paired-end sequencing on the Illumina HiSeq platform. After removing sequences with 3’ end adaptors and reads with an average quality below Q20, the clean data was aligned with the genome using Trinity software. The Genome-guided pattern of Trinity was employed for *de novo* assembly and clustering based on alignment results. The longest transcript was selected as the unigene and used for gene functional annotation and gene structure prediction. Gene function annotation was conducted by comparing gene sets identified through gene structure annotation using InterProScan5 with established protein databases such as SwissProt, NR, Pfam, KEGG, and InterPro. The annotated species included *Caenorhabditis elegans*, *Leishmania donovani*, *Reticulomyxa filosa*, *Paramecium duboscqui*, *Tetrahymena thermophila*, *Oxytricha trifallax*, *Stylonychina lemnae*, *Euplotes vannus*, *Euplotes octocarinatus*, and *Euplotes aediculatus*.

### Prediction of cilia-associated gene families in *E. amieti*

Cilia-associated genes were identified by referencing the reported cilia and centrosomes genes database (Arnaiz et al., 2009).^1^ Relevant sequences of cilia-associated genes were downloaded from the NCBI database using the BLAST similarity retrieval system. Utilizing sequence similarity alignment, the downloaded cilia and centrosomes genes sequences were employed as queries to identify homologous genes within the *E. amieti* genome by applying BLASTP software (*E*-value = 1e-5). The NR function and Pfam domain of these predicted protein sequences were annotated using BLASTP. For the genetic comparison among various hypotrich ciliates, genomic data for four additional hypotrich ciliates were retrieved from genome databases for *Euplotes vannus*,^2^ *Euplotes octocarinatus*,^3^ and *Oxytricha trifallax*,^4^ and *Stylonychia lemnae*.^5^

### Bioinformatics analysis of cilia-associated gene families

Gene annotation, metabolic pathway annotation, gene function annotation, protein classification, and disease-associated annotation were conducted using various online tools, including NR,^6^ WebGestalt,^7^ METASCAPE,^8^ and GO.^9^ Conserved protein domains were analyzed using MEME^10^ online software, while Pfam^11^ was used for protein domain prediction. Protein interactions were analyzed using the STRING database (PPI enrichment *p*-value < 1.0e-16). Genetic disease networks were visualized and analyzed using the DisGeNET database. Data visualization tools, including Venn diagrams, chord diagrams, and enrichment dot bubbles, were created using Origin Pro 2024b.

Screening and bioinformatics analysis of central genes The FASTA sequences of ciliopathy proteins obtained via BLAST software were imported into the STRING database^12^ to create PPI networks. These network diagrams were further optimized and analyzed using Cytoscape software.^13^ The topological parameters of each protein in the PPI network were calculated using 12 topological analysis methods available in the CytoHubba plug-in of Cytoscape. The top 50 proteins, based on differential expression genes (DEGs) parameters from the 12 algorithms, were selected, and those appearing in the top 50 of at least six algorithms were identified as pivotal hub genes.

### Identification of frameshifting genes

The identification of frameshifting genes was conducted following the methodology outlined by Wang (2017). Initially, all cilia-associated genes transcripts were aligned using BLASTX against the NCBI’s non-redundant protein database (*E*-value = 1e-5). Subsequently, transcripts that aligned to the same protein sequence but exhibited different open reading frames were identified and analyzed.

Based on the reported Euplotes PRF genes, the initial ORF consistently terminates with a 5′-AAA TAR-3′sequence. The Euplotes stop codon, either TAA or TAG was identified at the end of the starting reading frame. Subsequently, the “T” or “TA” was excised from the stop codon, contingent upon the potential type of frameshift. The resulting frameshift genes (fsgenes) were re-evaluated against the non-redundant protein database of NCBI. Once the C-terminal extended protein was produced as a results of the frameshift, the gene was classified as a PRF gene.

### PCR and qPCR

Cell division in *E. amieti* was induced by synchronized post-starvation feeding, with 80% of the cells entering the division phase 8 h after feeding on *Chlorogoium elongatum*. RNA extraction from *E. amieti* in both the division and G0 phases was performed using the RNeasy kit, following the manufacturer’s instructions. cDNA synthesis was conducted using the PrimeScrip™ RT Master Mix kit (Code No. RR036A, TaKaRa, Japan). Amplification was carried out using the TaKaRa PCR reaction kit. The reaction conditions included an initial denaturation at 94°C for 5 min, followed by 30–45 cycles of denaturation at 94°C for 45 s, annealing at 46°C for 45 s, and extension at 72°C for 1 min. A final extension at 72°C for 10 min was followed by storage at 4°C.

Quantitative PCR (qPCR) was performed using TB Green™ Premix Ex Taq™ II (Tli RNaseH Plus) (Takara Bio, Inc.) on samples from three independent experiments, following the manufacturer’s guidelines. Thermocycling conditions were pre-denaturation at 95°C for 30 s, followed by 35 cycles of denaturation at 95°C for 10 s, and annealing and extension at 60°C for 30 s. All experiments were conducted in duplicate, using the GAPDH gene as the housekeeping gene for normalization and quantification. The relative fold change in expression was calculated using the 2^−ΔΔ*C*_q_^ method [Δ*C*_q_ = *C*_q_ (target gene)−*C*_q_ (reference gene), ΔΔ*C*_q_ = ΔC_q_ (target sample) −Δ*C*_q_ (control sample)], with *C*_q_ values below 30 considered valid. Data analysis followed the manufacturer’s guidelines. Primer sequences are listed in Supplementary Table S1.

### RNA interference by feeding method

Following the methodology outlined by Paschka et al. (2003), *E. coli* HT115 strains harboring recombinant expression vectors *L4440-ARL2BP* and *L4440-DYNLRB2* were used to feed *E. amieti*. The L4440 vector is a widely used plasmid for RNAi studies, particularly in ciliates, due to its ability to express dsRNA under the control of the T7 promoter. The control group was fed *E. coli* HT115 containing the empty L4440 vector without target gene fragments to rule out non-specific effects of dsRNA expression. This control allowed us to rule out non-specific effects of dsRNA expression on cell viability and motility, ensuring that the observed phenotypic changes were specifically due to the silencing of *ARL2BP* and *DYNLRB2*. *E. coli* HT115 strains containing L4440, *L4440-ARL2BP*, and *L4440-DYNLRB2* were diluted at a 1:100 ratio and inoculated into LB liquid culture medium, incubated at 37°C with shaking at 210 rpm for 180 min. Upon reaching an *OD*_600_ of 0.4, isopropyl IPTG was added to a final concentration of 0.4 mM to induce dsRNA expression for 3 h. The bacteria were then washed three times with 1 mL of double-distilled water. Finally, 30 μL of *E. coli* HT115 was fed to every 2 × 10^6^ cells.

### FLUTAX staining

Cells were fixed in 1 mL of 4% paraformaldehyde at room temperature for 10 min and washed three times in ice-cold 0.1 M phosphate-buffered saline (PBS) (5 min per wash). Cells were then incubated in Flutax-2 (Oregon Green™ 488 conjugate, Invitrogen, United States) for 10 min at room temperature without permeabilization. After incubation, cells were washed briefly and mounted with 15 μL Slowfade Gold Antifade Mountant with DAPI (Thermo Fisher Scientific, United States). Images were acquired using an Olympus IX81 fluorescence microscope (Olympus, Japan).

### Determination of swimming speed and swimming curve of *E. amieti*

According to the method of Arnaiz et al. (2009), 4–8 *E. amieti* were transferred onto a glass slide and observed under a microscope (IX81, Olympus). Images were captured at 0.3-s intervals under a 10 × objective lens and analyzed using Prism and ImageJ software.

### Statistical methods

Quantitative data with a normal distribution were presented as means ± standard error of the mean. Two independent sample *t*-tests were used to compare the two groups. Statistical analysis was performed using GraphPad Prism software, with a significance level set at *p* < 0.01 considered statistically significant.

## Results

### *E. amieti* is an ideal model for studying cilia-associated genes

The application of next-generation sequencing technologies to sequence and analyze the macronuclear genome of *E. amieti* has provided an in-depth understanding of its genetic composition. The gene annotation of genome sequencing of E. amiteti has been uploaded to the NCBI database (). The genome of *E. amieti*, which is 89 Mb in size with a GC content of 33%, aligns with the typical characteristics observed in ciliates, known for their elevated AT content. The identification of 105 rRNA genes and a substantial gene count of 27,650 highlights the intricate and diverse nature of the *E. amieti* genome. Particularly intriguing is the reassignment of the stop codon UGA to encode cysteine (Cys), a feature it shares with *Euplotes octocarinatus* and *Euplotes vannus*, highlighting a unique aspect of genetic code evolution within the *Euplotes* genus (Table 1).

**TABLE 1:** Comparison of representative ciliate macronuclear genomes.

	Assembly size (Mb)	GC (%)	Number of tRNA genes	Number of genes	Stop codon reassignment
*Euplotes amieti*	89	33	105	27,650	UGA/Cys
*Paramecium duboscqui*	28.82	34	–	16,774	UAR/Gln
*Tetrahymena thermophila*	104	28	715	27,424	UAR/Gln UGA/Ser
*Euplotes vannus*	85	37	109	43,040	UAR/Cys
*Euplotes octocarinatus*	89	28	95	29,076	UGA/Cys
*Oxytricha trifallax*	50	31	93	18,400	UAR/Gln
*Stylonychia lemnae*	50	31	50	12,000	UAR/Gln

This table presents the characteristics of several representative ciliate macronuclear genomes, including assembly size (in Mb), GC content (%), number of tRNA genes, total number of genes, and stop codon reassignment.

To validate the sequencing results, a comparative analysis was conducted with other ciliate genomes, including *Paramecium duboscqui*, *Tetrahymena thermophila*, *Oxytricha trifallax*, *Stylonychia lemnae*, *Euplotes vannus*, and *Euplotes octocarinatus*. The findings indicate a higher number of successfully annotated genes in the *E. amieti* genome compared to other ciliates, suggesting potential unique gene features or the influence of increased sequencing depth. Additionally, these comparisons reveal variability in the evolution of the genetic code among ciliates, with *E. amieti* exhibiting genomic similarities to other *Euplotes* species, particularly in genome size, GC content, and the reassignment of UGA-encoded cysteines. These findings suggest a close evolutionary relationship and potentially similar biological functions and ecological roles in their respective environments. Consequently, the annotation and identification of the *E. amieti* genome align with existing knowledge of ciliates, providing additional genetic insights.

**FIGURE 1:**
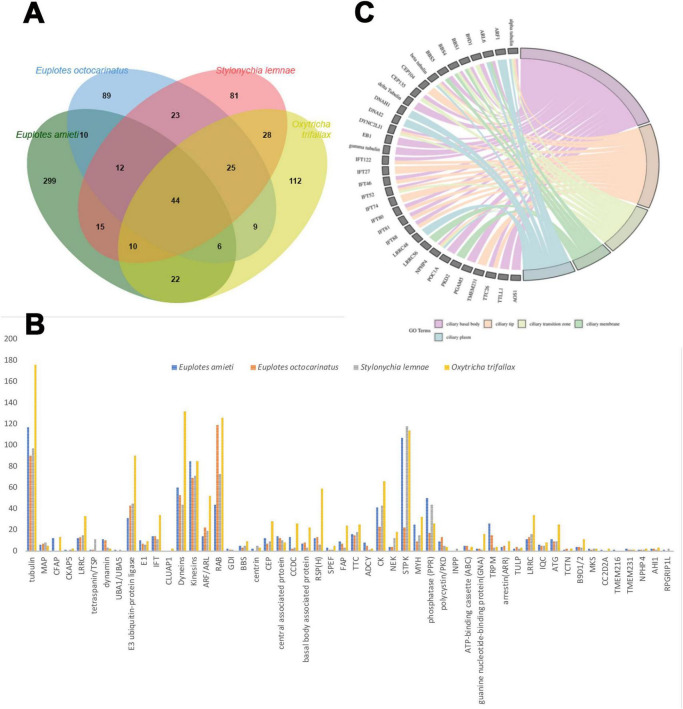
Comparison of the cilia-associated genes families among four hypotrich ciliates. **(A)** Venn diagram illustrating the gene overlap among four species of hypotrich ciliates: *E. amieti*, *Euplotes octocarinatus, Stylonychia lemnae*, and *Oxytricha trifallax*. Each oval corresponds to the gene set of a specific species, with numerical values denoting the number of genes that are either unique to that species or shared among multiple species. The bar graph displays the total number of genes analyzed for each species. The diversity of cilia-associated protein family members among four distinct species of hypotrich ciliates: *E. amieti* (blue), *Euplotes octocarinatus* (orange), *Stylonychia lemnae* (gray), and *Oxytricha trifallax* (yellow). The x-axis lists the cilia-associated proteins, while the y-axis denotes the count of gene family members. **(B)** The cilia-associated gene families in four hypotrich ciliates. The numbers in the y-axis indicate the number of genes to be classified in the gene families. **(C)** The GO chord demonstrates the localization enrichment of cilia-associated proteins that are conserved across four different species of hypotrich ciliates, namely *E. amieti*, *Euplotes octocarinatus, Stylonychia lemnae, and Oxytricha trifallax*. The color-coded key indicates the subcellular localization of these proteins within the cilia, with pink representing the ciliary basal body, orange indicating the ciliary tip, pale green denoting the transition zone, green representing the ciliary membrane, and blue indicating the ciliary plasma.

To elucidate the genetic components associated with cilia, 418 cilia-associated genes were identified in *E. amieti* by referencing human and reported cilia and centrosomes genes in the Cildb database. In hypotrich ciliates, dorsal-ventral differentiation influences the organization and clustering of cilia in the dorsal and ventral cortex, necessitating further investigation into cilia-associated genes to elucidate their unique ciliary assembly traits. This study undertakes a comparative analysis of cilia-associated genes in *E. amieti* with three other recognized hypotrich ciliates. Among the identified genes, 299 newly annotated cilia-associated genes are unique to *E. amitei* (Figure 1A; Supplementary Table S1). All analyzed cilia-associated genes in the four hypotrich ciliates include 52 gene families (Figure 1B; Supplementary Table S2). Notably, families such as tubulin, dyneins, kinesins, ARF (ADP-Ribosylation Factor), and STPK (Serine/Threonine Protein Kinase) have more than 50 members. The number of members within these protein families varies significantly across different species. Tubulin, a microtubule protein, is abundant in all four ciliate species, indicating its crucial role in the structure and function of cilia. *Oxytricha trifallax* expresses a higher number of dyneins and RAB proteins (Ras-related in brain proteins) compared to the other three species, suggesting a potential specialization or increased reliance on dynein-mediated processes and vesicular transport or signaling pathways.

Common cilia-associated genes across all four ciliates include the tubulin, dynein, and intraciliary transporter families. *E. amieti* shows higher expression of tubulin, kinesins, and dyneins, which are essential for ciliary movement and assembly. These proteins typically contribute to the structural integrity and motility functions of cilia. In contrast, *Euplotes octocarinatus* has higher expression levels of proteins such as centrin, CEP (centrosomal protein), and RSPH (radial spoke head protein). Centrin is involved in centriole stability, CEP proteins in centrosome and cilia function, and RSPH in ciliary beat regulation. Furthermore, 44 cilia-associated genes were found to be conserved across the four types of hypotrich ciliates, indicating the high conservation of these genes in hypotrich ciliates (Figure 1A). The subcellular localization of gene products in all four ciliates was enriched in five parts of the cilia: the ciliary basal body, transition zone, plasma, membrane, and tip (Figure 1C). These findings suggest that *E. amieti* can serve as a valuable model organism for investigating ciliopathies.

### Function annotation of cilia-associate genes in *E. amieti* genome

**FIGURE 2:**
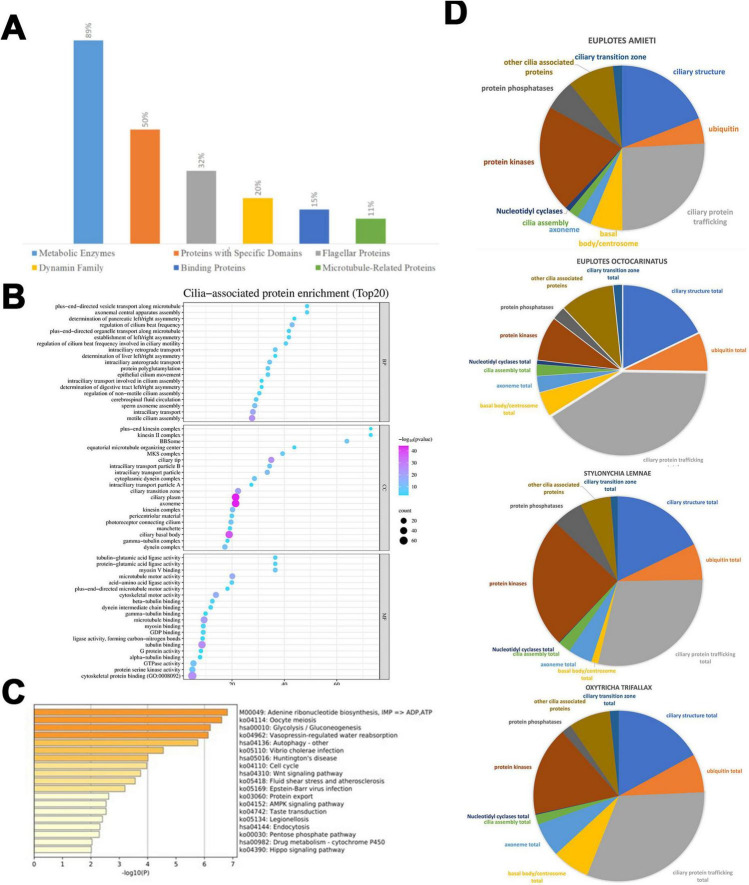
Functional differences among four hypotrich ciliates. **(A)** NR function annotation of predicted cilia-associated genes. The distribution of segments reveals that enzymes constitute the Metabolic Enzymes at 89%, Proteins with Specific Domains at 50%, Flagellar Proteins at 32%, Dynamin Family at 20%, binding proteins at 15%, and Microtubule-Related Proteins at 5%. **(B)** This enrichment dot bubble visualizes the top 20 enriched biological processes, cellular components, and molecular functions associated with cilia. Each point represents a functional category, with the size of the point indicating the number of proteins (count) in that category, and the shade of the point reflecting the negative logarithm of the *p*-value [- log(*p*-value)], where a pink/purple end of the scale signifies higher statistical significance. The x-axis represents the “count,” indicating the number of proteins associated with each term, while the y-axis lists various GO terms related to cilia. The size of each bubble corresponds to the protein count, and the color intensity signifies the level of enrichment, with pink/purple end of scale indicating a lower *p*-value (higher significance) and light blue end of scale indicating a higher *p*-value (lower significance). **(C)** Pie charts illustrate the variation in genes with different cilia-associated functions across different ciliate species, namely *E. amieti*, *Euplotes octocarinatus*, *Stylonychia lemnae*, and *Oxytricha trifallax*. Functions associated with cilia are divided into the ciliary transition zone, ciliary structure, ciliary protein trafficking, basal body/centrosome, ubiquitin, nucleotidyl cyclases, cilia assembly, protein kinases, protein phosphatases, and other cilia-associated proteins. **(D)** The horizontal bar chart represents the significance of various biological pathways in the cilia-associated proteins of *E. amieti* based on a pathway enrichment analysis. Each bar corresponds to a specific pathway, with the pathway names and their respective KEGG identifiers listed on the right. The length of each bar indicates the negative logarithm of the *p*-value [- log10(*p*)] for the enrichment of that pathway, with longer bars representing higher significance (lower *p*-value).

GO enrichment analysis of the predicted cilia-associated genes was performed using the MATESCAPE online analysis platform (Figure 2A). The results indicate that the majority of these genes encode enzymes involved in metabolic processes, such as those related to glucose metabolism, including phosphoglycerate mutase, malate dehydrogenase, and ATPase, which are crucial for energy metabolism, comprising 89% of the total. Additionally, 50% of these genes encode proteins containing specific domains, such as the protein kinase domain, tetratricopeptide repeat domain, and adenylate kinase domain. Furthermore, 32% of the genes belong to the family of flagellar proteins, including intra-flagellar transport proteins and flagellar/matrix proteins. Moreover, 20% of the genes are part of the dynamin family, encompassing dynein intermediate chain, dynein heavy chain, and dynein light chain. Another 15% of the genes are categorized within the binding protein family, which includes actin-binding proteins and ran-binding proteins, while 11% fall under the microtubule-related protein family (e.g., tubulin ε chain, α-tubulin N-acetyltransferase). A minority of the proteins are associated with transport or autophagy.

The top 20 enrichments from the GO analysis reveal the primary functional categories of the cilia-related proteins. Within the Biological Process (BP) category, the top three functions include plus-end-directed vesicle transport along microtubules, axonemal central apparatus assembly, and determination of axonemal central apparatus assembly. In the Cellular Component (CC) category, the predominant entries are the ciliary membrane, axoneme, and pericentriolar material. The top three functions in the Molecular Function (MF) category are tubulin binding, microtubule motor activity, and dynein light intermediate chain binding (Figure 2B).

In addition, KEGG pathway enrichment analysis of the predicted cilia-associated genes using the MATESCAPE platform revealed significant enrichment in pathways such as adenine ribonucleotide biosynthesis, oocyte meiosis, glycolysis/gluconeogenesis, vasopressin-regulated water reabsorption, Wnt signaling pathway, AMPK signaling pathway, and Huntington’s disease (Figure 2C).

### Comparison of cilia-associated genes in four hypotrich ciliate

The cilia-associated genes were categorized into 11 classes based on their roles with cilia (Figure 2D). The composition of these classes varies among the four ciliates studied. In *E. amieti*, the most prominent functional categories include ciliary structure, other cilia-associated proteins, and ubiquitin. Conversely, in *Euplotes octocarinatus*, the largest categories are ciliary structure, ciliary transition zone, and other cilia-associated proteins. For *Stylonychia lemnae*, the ciliary structure, ciliary transition zone total, and basal body/centrosome total are the most prominent categories. In *Oxytricha trifallax*, the dominant categories include ciliary structure, ciliary transition zone total, and other cilia-associated proteins.

### Structure characterization of cilia-associated genes and their encoded proteins

**FIGURE 3:**
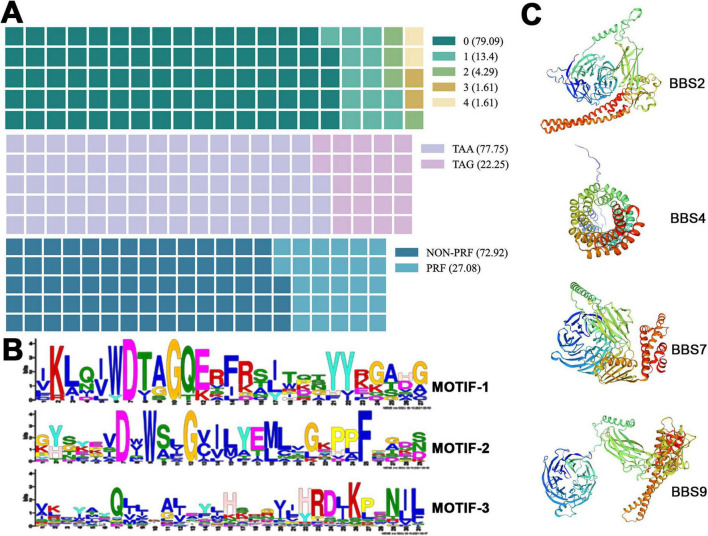
Characteristics of cilia-associated genes in *E. amieti.* **(A)** The waffle chart represents the percentage of each structural characteristic of cilia-associated genes. The upper indicates the proportion of genes containing different numbers of introns (green to light yellow). Number 0∼4 indicate the numbers of introns. The middle indicates the proportion of genes with different stop codons (purple to pink). The lower indicates the proportion of genes containing PRF (blue to light blue). The numbers in parentheses represent the proportion. **(B)** Three motifs shared among these cilia-associated genes in *E. amieti*. The frequency of occurrence of letters in a sequence is represented by their relative size, with the height of each letter being proportional to the frequency of the corresponding base at that position, typically measured in bits. The arrangement of letters at each position from largest to smallest based on conservation allows for easy identification of conserved sequences by examining the top letters. **(C)** The three-dimensional structures of four distinct BBS proteins with PRF genes, namely BBS2, BBS4, BBS7, and BBS9. A unique color scheme highlights the secondary structure elements, including alpha helices and beta sheets. These proteins in the same BBS family contain similar wreath-like domains.

The structural features of the cilia-associated genes in *E. amieti* were analyzed. Approximately 79.09% of these genes lacked introns, 13.4% contained one intron, and genes with multiple introns accounted for less than 10% of the total. All the genes utilized either TAA or TAG as the stop codon, with over three-quarters employing TAA and less than one-quarter utilizing TAG. Furthermore, around 30% of these genes undergo programmed frameshifting (Figure 3A). Localization analysis revealed that the majority of the gene products were found in the basal body, with 40 proteins localized in basal bodies encoded by PRF genes. The predominant + 1 programmed ribosomal frameshift (PRF) was observed, with AAATAA identified as the slippery sequence (Table 2).

**TABLE 2:** Overview of protein frameshifting and cellular localization.

Protein	Slippery sequence	Frameshifting model	Numbers of frameshfting	Cellular localization
BBS2	AAATAA/AAATAA	+1	2	Ciliary basal body
BBS4	AAATAA	+1	1	Ciliary basal body
BBS7	AAATAA/AAATAG/AAATAA	+1/+1/+1	3	Ciliary basal body
BBS9	AAATAA/AAATAA	+1/+1	2	Ciliary transition zone/centrosome
IFT88	TGTTAG	+2	1	Ciliary basal body/transition zone/ciliary plasm/ciliary tip
IFT122	AATTAA	+1	1	Ciliary basal body/ciliary transition zone/ciliary tip
IFT140	AAATAA/AAATAA	+1/+1	2	Ciliary basal body/transition zone/ciliary plasm/ciliary tip
CEP104	AAATAG	+1	1	Ciliary transition zone/centriole
CEP131	AAATAA	+1	1	Ciliary basal body/ciliary transition zone
CEP135	AAATAA	+1	1	Ciliary basal body/ciliary transition zone
CEP290	AAATAA	+1	1	Ciliary basal body/ciliary transition zone
CCDC151	AAATAA	+1	1	Ciliary basal body/ciliary tip
CFAP36	AAATAA	+1	1	Ciliary transition zone/ciliary base
CFAP52	AAATAA	+1	1	Ciliary basal body/ciliary plasm
γ-tubulin	TTCTAA/TATTAA	+1/+1	2	Ciliary basal body/transition zone/ciliary plasm/ciliary tip
δ-tubulin	AAGTAA	+1	1	Ciliary basal body/transition zone/ciliary plasm/ciliary tip
ε-tubulin	TGATAG	+1	1	Ciliary basal body/transition zone/ciliary plasm/ciliary tip
TTLL11	AAATAA	+1	1	Ciliary basal body
CC2D2A	AAATAA/AAATAA	+1/+1	2	Ciliary transition zone
KIF2A	AAATAA	+1	1	Cilium/centrosome
KIF13	AAATAA	+1	1	Centrosome
BBOF1	GGATAA	+1	1	Ciliary basal body
DYNC2H1	ATATAG	+1	1	Ciliary base/ciliary tip
CDKF-4	AAATAA	+1	1	Ciliary basal body
Dynactin	CGATAA	+1	1	Ciliary basal body
LRRC49	AAATAA	+1	1	Centrosome
SPEF1	AAATAA	+1	1	Ciliary basal body
WDR35	AAATAA	+1	1	Ciliary basal body/ciliary plasm/ciliary tip
RANBP1	AGTTAA	+1	1	Centrosome
VPS35	TTTTAG	+2	1	Centriole/ciliary basal body organization
SSNA1	TTTTAG	+2	1	Ciliary basal body
CKAP5	AAATAA/AAATAA/AAATAA	+1/+1/+1	3	Ciliary basal body/ciliary plasm/ciliary tip/transition zone
SPC97/98	ATATAA	+2	1	Centrosome

This table summarizes the characteristics of various proteins associated with ciliary structures, focusing on the slippery sequences, frameshifting models, the number of frameshifts, and their cellular localization.

Visual analysis of conserved domains of proteins encoded by cilia-associated genes was conducted using the MEME online software. Three conserved sequences were identified (Figure 3B). Among the 418 cilia-associated proteins analyzed, 37 were found to contain these conserved sequences (ARL3, ARL2, RAB11B, RABL2A, DNAL1, RAB6B, RAN, RAB8B, RAB1A, MAPK15, CSNK1E, AKT3, MDH1, STK38L, CAMK2D, PRKACB, ICK, MAP3K2, GSK3B, MOK, SLC47A1, PLK1, NEK1, RAB2A, PRPF4B, KIF9, NEK4, CDK1, KIF3B, AK7, STK36, CFAP65, PIK3R4, MARK4, PI4KA). These proteins are annotated as “cilia-associated” based on their gene origins, suggesting potential roles in ciliary functions. However, subcellular localization data from existing studies (e.g., UniProt) indicate that some members (e.g., RAN, AKT3) may also localize to the nucleus or cytoplasm, implying multifunctional roles beyond cilia. Further experimental validation is required to confirm their exclusive ciliary localization. The distribution of these sequences revealed that Motif-1 was the most prevalent, present in 26 proteins, followed by Motif-2 in 12 proteins, and Motif-3 in 10 proteins. Additionally, 11 proteins were identified to contain two distinct domains. It is hypothesized that proteins containing different domains may exhibit variations in functionality and evolution. For structural prediction, the BBS (Bardet-Biedl Syndrome) family proteins, known for their localization to basal bodies and close relation to cilia assembly, were selected. The predicted structures of BBS family proteins BBS2, BBS4, BBS7, and BBS9 are depicted in Figure 3C. Notably, BBS2, BBS7, and BBS9 share common conserved motifs, including leucine zipper and a basket structure formed by β-sheets.

### Screening and analysis of hub genes in the cilia-associated gene families

**FIGURE 4:**
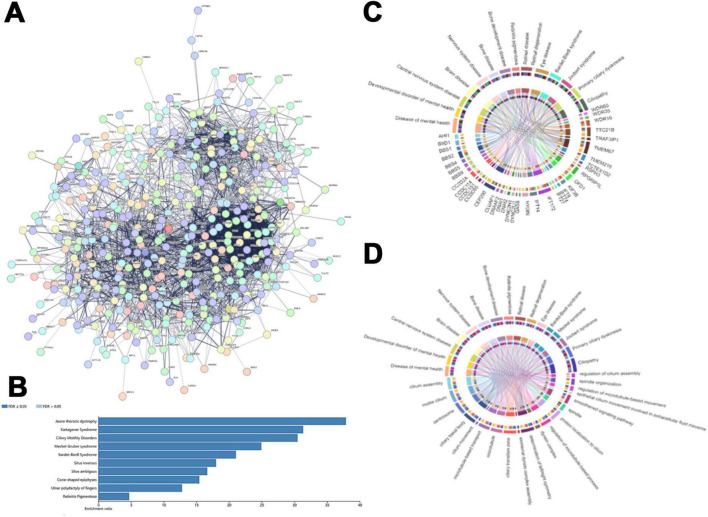
The central genes in the cilia-associated gene families. **(A)** The protein functional interaction network of ciliates. Each circle indicated one protein. **(B)** The horizontal bar chart that shows the enrichment ratio of ciliopathy. Each bar represents a different condition, with the condition names listed on the left side of the chart. The length of the bars corresponds to the enrichment ratio. **(C)** Correlation between the hub cilia-associated genes and ciliopathy phenotype. The upper rim of the circle denotes the ciliopathy phenotypes, while the lower rim represents distinct hub cilia-associated genes. **(D)** The circos plot illustrates the intricate network of relationships between biological processes of cilia and cilia-related diseases. The upper rim of the circle indicates the cilia-related disease labels and the lower rim represents different biological processes of cilia. Interconnecting lines within the circle depict the interactions or correlations between these entities, with the arrangement and density of lines indicating the level of interconnectivity. Colored concentric circles within the plot represent additional quantitative information or categorization.

An interaction network of 418 cilia-associated genes in *E. amieti* was constructed using the STRING database, illustrating direct or indirect interactions among these genes (Figure 4A). Within this network, 39 hub genes were identified, including *WDR60*, *IFT88*, *IFT122*, *IFT140*, *IFT172*, *WDR35*, *IFT81*, *DYNLL2*, *DYNC2H1*, *DYNC2LI1*, *WDR19*, *IFT80*, *IFT22*, *IFT27*, *KIF3B*, *VCP*, *IFT74*, *BBS1*, *BBS2*, *BBS4*, *TTC26*, *IFT57*, *BBS5*, *BBS9*, *OFD1*, *TTC21B*, *IFT46*, *CEP290*, *CLUAP1*, *HSPA8*, *TRAF3IP1*, *DNAI2*, *CCDC65*, *CCT3*, *RSPH3*, *SPAG6*, *ACTB*, *CLTC*, and *DYNC1H1*. To further investigate the function of these hub genes, their relationships with ciliopathies were explored using DisGeNET, a tool for analyzing genetic disease networks (Piñero et al., 2017). The analysis revealed that these hub genes are involved in various ciliopathies, with the top 10 including Jeune thoracic dystrophy, Kartagener syndrome, ciliary motility disorders, Meckel-Gruber syndrome, Bardet-Biedl syndrome, situs inversus, situs ambiguous, cone-shaped epiphyses, ulnar polydactyly of fingers, and retinitis pigmentosa (Figure 4B). These hub genes are linked to ciliopathic phenotypes involving the central nervous system, brain diseases, skeletal development disorders, retinal diseases, BBS syndrome, Joubert syndrome, Meckel syndrome, and primary ciliary dyskinesia. The relationship between hub genes and ciliopathic phenotypes was visualized using a GO chord diagram. The study found that *SPAG6*, *TRAF3IP1*, *IFT22*, and *KIF3B* showed lower correlations with ciliopathies, while *IFT172*, *TTC21B*, *CEP290*, *ACTB*, and *DYNC1H1* were highly correlated. This suggests that certain genes are involved in multiple ciliopathies, while others are specific to individual types (Figure 4C).

Our analysis delved into the cellular components, molecular functions, and biological processes involved in these ciliopathies linked to the hub genes (Figure 4D). The GO enrichment revealed that the primary cellular components include cilia assembly, ciliary motility, centrosomes, and cilia basal bodies, as well as microtubule-based transport, microtubules, and cilia transition zones. The molecular functions and biological processes include the axial filament dynamic protein complex assembly, left/right symmetry determination, regulation of dynein complexes, regulation of microtubule processes, and protein localization to cilia. The correlation between ciliopathies and MF, BP, and CC suggests that ciliary assembly, motile cilia, ciliary basal bodies, and centrosomes are related to various types of ciliopathies. Conversely, factors such as abnormal ciliary beating patterns, extracellular fluid dynamics, and certain dynein-related processes (e.g., non-axonemal dynein assembly) are currently thought to play less direct roles in the pathogenesis of classical ciliopathies, though their contributions may vary across specific disease subtypes.

### Expression and functional validation of some cilia-associated genes in *E. amieti*

**FIGURE 5:**
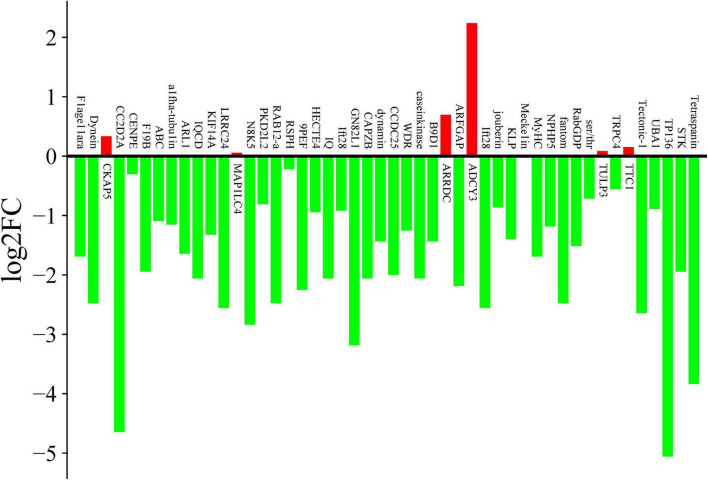
qPCR expression changes of cilia-related genes during cell division. The vertical axis is log2FC (log2 fold change), green indicate the down-regulated genes, and red indicates the up-regulated genes. The number in the vertical axis indicate the fold of the change in expression.

In ciliates, ciliogenesis exhibits a strong correlation with morphogenesis during the division phase. Extensive research has demonstrated that the dorsal and ventral cilia of the mother cells undergo replication prior to division and are systematically allocated to both daughter cells during division. Consequently, investigating the function of ciliary-associated proteins necessitates an initial examination of ciliary proteins that are regulated by the cell cycle. To verify the expression levels of cilia-associated genes in *E. amieti*, 48 genes were randomly chosen from the 52 cilia gene families for measurement using qPCR, comparing levels to those in the G0 phase. The findings revealed that the majority of the selected cilia-associated genes were downregulated during cell division. In contrast, six genes showed significant upregulation, including *CKAP5*, *MAP1LC4*, *ARRDC*, *ADCY3*, *TULP3*, and *TTC*, with *ADCY3* (adenylate cyclase type 3) showing the highest level of upregulation (Figure 5).

**FIGURE 6:**
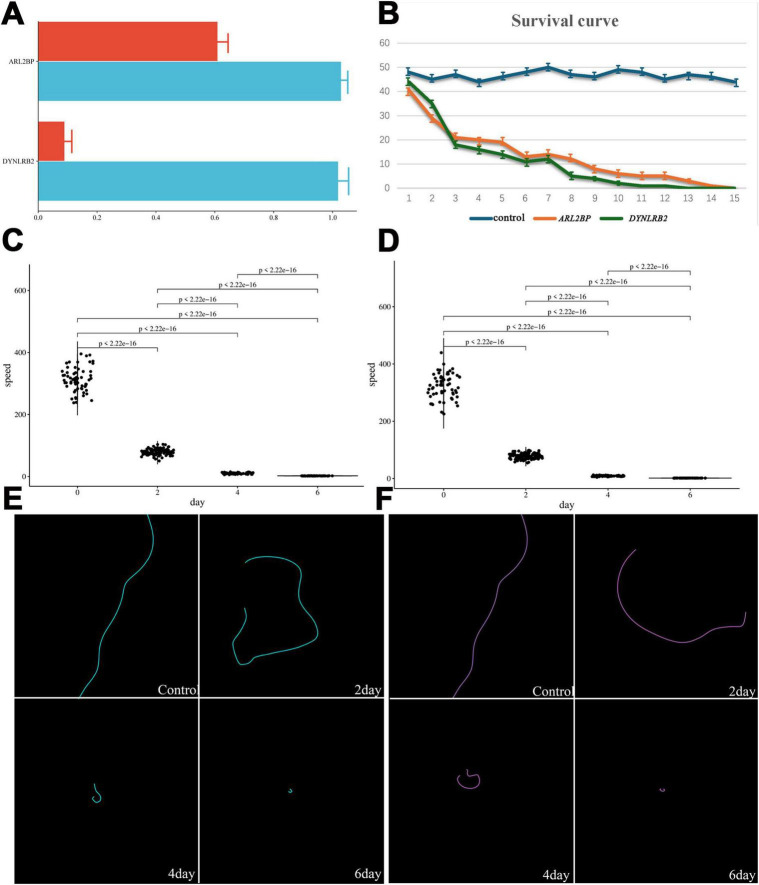
*ARL2BP* and *DYNLRB2* interference verification and changes in *E. amieti.* **(A)** The interference efficiency of *ARL2BP* and *DYNLRB2* was tested by RT-PCR after RNA interference by feeding method, red is the interference group, and blue is the control group. **(B)** The survival curves of control (blue), *ARL2BP* (orange), and *DYNLRB2* (green), respectively. **(C)** The movement speed of *ARL2BP* changes with the number of days of interference, the horizontal axis is the number of days of interference, and the vertical axis is the movement speed. **(D)** The movement speed of *DYNLRB2* changes with the number of days of interference, the horizontal axis is the number of days of interference, and the vertical axis is the movement speed. **(E)** The movement trajectory of *ARL2BP* with the number of days of interference (blue). **(F)** The movement trajectory of *DYNLRB2* with the number of days of interference (green).

To confirm the connection between the 418 cilia-associated genes and ciliary assembly, we selected *ARL2BP* and *DYNLRB2* for functional validation. These genes were chosen based on their high expression levels in cilia-associated structures and their known roles in ciliary assembly and motility. We hypothesized that silencing these genes would disrupt ciliary function, leading to reduced motility and altered cytoskeletal organization in *E. amieti*. *ARL2BP* is a protein present in the basal body regions of photoreceptor cells and is needed for normal photoreceptor cilia doublets and outer segment structure. *DYNLRB2* is a microtubule motor protein complex that is responsible for the retrograde transport of cellular cargoes, and is necessary for mitotic regulation. The amplified target fragments of *ARL2BP* and *DYNLRB2* were inserted into L4440 to create the recombinant plasmids pL4440-*ARL2BP* and pL4440-*DYNLRB2*. The expression of dsRNA of the target genes in *E. amieti* was verified using qPCR. The findings indicated that the inserted gene fragments exhibited significant expression in pL4440-*ARL2BP* and pL4440-*DYNLRB2* compared to the control group. Following the feeding of bacteria containing the interference expression vector, the relative expression levels of *ARL2BP* and *DYNLRB2* mRNA were notably decreased in the interference group compared to the control group (Figure 6A). These results demonstrate that RNA interference targeting *ARL2BP* and *DYNLRB2* significantly reduces their expression levels, leading to observable phenotypic changes such as increased mortality and reduced motility in *E. amieti*. This confirms the functional importance of these genes in ciliary assembly and motility.

The impact of RNAi on cell viability, motility, and cytoskeleton was further investigated. Analysis of the growth curve revealed a significant decrease in the number of surviving *E. amieti* over time following RNAi treatment compared to the control group. By the 10th day, the population of surviving *E. amieti* was notably diminished, and by day 15, all *E. amieti* had perished (Figure 6B).

The swimming trajectories and speeds of *E. amieti* following interference with *ARL2BP* or *DYNLRB2* at 2, 4, and 6 days were also analyzed. In the control group, *E. amieti* exhibited typical linear swimming patterns. However, at 2, 4, and 6 days post-*ARL2BP* interference, *E. amieti* exhibited circular swimming trajectories with decreasing radii over time, eventually leading to a stationary spinning motion by days 4–6 (Figures 6C,D). Additionally, a significant decrease in swimming speed was observed with prolonged interference (Figures 6E,F).

**FIGURE 7:**
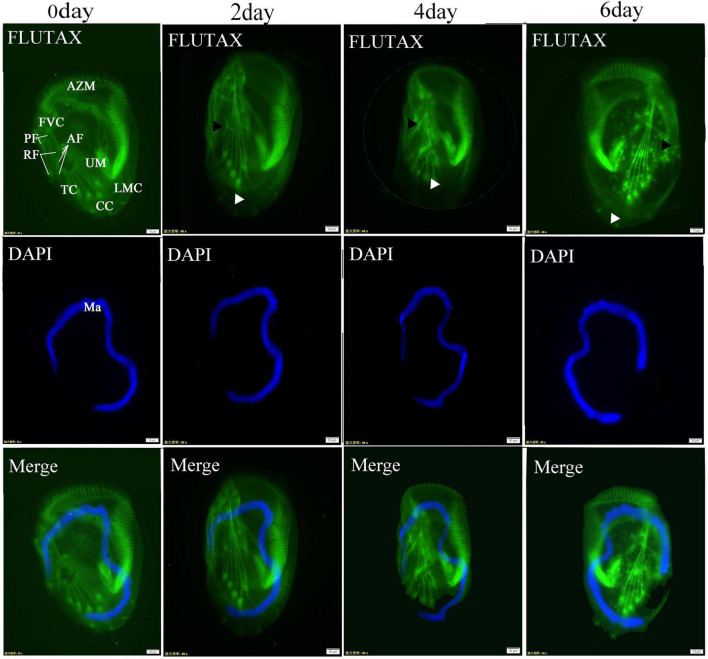
Post-interference immunofluorescence. *ARL2BP* immunofluorescence, blue is the marker for the cell nucleus, green is the marker for the cytoskeleton; AZM, adoral zone of membranelles; PM, paroral membranes; FVC, frontoventral cirri; TC, transverse cirri; LMC, left marginal cirri; CC, caudal cirri; Dks, Dorsal Kineties; Ma, macronucleus; AF, anterior longitudinal fiber; PF, the posterior longitudinal fiber; RF, the radiating fiber. The black triangles indicate the microtubules associated with frontoventral cirri, and the white triangles indicate the caudal cirri. Bar = 20 μm.

The organization of cilia and the cytoskeleton was observed using Flutax. The results revealed that the depletion of *ARL2BP* or *DYNLRB2* did not result in significant alterations in ciliary structure; however, notable changes were observed in the arrangement of cortical microtubules linked to the basal bodies following RNA interference of *ARL2BP*, exhibiting a curved and disordered pattern. Both the caudal cirri and the microtubules associated with frontoventral were observed to disappear (Figure 7).

## Discussion

The availability of numerous sequenced genomes from ciliated eukaryotes has set the stage for powerful genomic studies. It is currently estimated that the human ciliome comprises approximately 1,200 genes (van Dam et al., 2019). The ciliary database Cildb was established as a resource for accessing information on cilia and ciliopathies, including a comprehensive collection of predicted proteins from the genomes of 18 species commonly used in cilia, flagella, or centrosome studies, such as *Caenorhabditis elegans*, *Chlamydomonas reinhardtii*, *Drosophila melanogaster*, *Homo sapiens*, *Mus musculus*, *Paramecium tetraurelia*, *Rattus norvegicus*, *Trypanosoma brucei*, *Tetrahymena thermophila* (Soares et al., 2020; Valentine and Van Houten, 2021). However, there is a notable absence of genomic data for hypotrich ciliates in this database. Recently, databases have been developed for *Euplotes crassus*, *Euplotes aediculatus*, *Euplotes vannus*, and *Euplotes octocarinatus*, facilitating the search, analysis, and comparison of gene signatures within the *Euplotes* genus. Despite these advancements, there is a scarcity of data about genes specifically linked to distinct cellular structures or biological processes, making it challenging to quickly obtain information about cilia-associated genes through conventional retrieval methods.

In the current investigation, 418 cilia-associated genes were identified based on cilia proteomic information from Cildb, representing the first ciliome in hypotrich ciliates. The predicted numbers of cilia-associated genes for *Oxytricha trifallax*, *Stylonychia lemnae*, and *Euplotes octocarinatus* are 256, 238, and 218, respectively. Previous studies have shown that the number of cilia-associated genes varies widely between species, ranging from 200 to 1,000, with most organisms containing homologs of human cilia-associated genes (Geremek et al., 2011; Sigg et al., 2017; Li et al., 2004). In the flagellar axoneme of *Chlamydomonas*, nearly 450 ciliary proteins were identified (Kim and Tsiokas, 2011). The ciliome of *Tetrahymena thermophila* was elucidated through a translated genome, leading to the identification of 223 ciliary proteins (Smith et al., 2005). These results suggest that the 418 cilia-associated genes identified in *Euplotes* likely encompass most of its ciliary genes. It is undeniable that the pool of candidate cilia-associated genes in ciliates will continue to expand as sequencing depth increases. For example, investigating ciliopathies and their genetic mechanisms has expanded the known set of human cilia-associated genes (). Conclusively, the cilia-associated gene data from *E. amieti* will serve as foundational information for the ciliary database and contribute significantly to the study of the mechanisms underlying cilia and cytoskeleton assembly in hypotrich ciliates. Furthermore, although 299 genes specific to *E. amieti* have been identified in comparison to the other three hypotrich ciliates, additional investigation is necessary to uncover more cilia-associated genes that are unique to ciliates, as opposed to those that are homologous to human cilia-associated genes.

In this study, cilia-associated genes from four common hypotrich ciliates were retrieved and subjected to comparative analysis. The results show that only 44 genes are shared among these four hypotrich ciliates. This limited overlap may be attributable to the complex nature of cilia and the functional diversity observed across different biological settings. It has been documented that out of 55 cilia-centric high-throughput studies cataloged in Cildb (Arnaiz et al., 2009), only 21 genes are recurrent in over 20 studies (Nevers et al., 2017). A comparison of cilia genes in different species showed similar results, with 131 highly homologous cilia genes present in sea anemones, sea urchins, and choanoflagellates (Sigg et al., 2017). Additionally, different cilia-associated proteins are expressed in different cilia-related structures even within human cells; for example, 868 proteins were identified in plexus epithelial cells, but only 152 were shared with the proteome of motile cilia and flagella (Narita et al., 2012). In the present study, despite *E. amieti* and *E. octocarinatus* being freshwater Euplotes with close phylogenetic relationship, there is a substantial disparity in the number of cilia-associated genes between the two species. This discrepancy may be attributed to variations in sequencing depth, and the annotation levels. Furthermore, distinguishing between members of the same gene family across different Euplotes presents significant challenges. The results of BLAST revealed that a substantial number of homologous sequences exist among members of various gene families, complicating the classification of specific genes into appropriate families due to ambiguous gene function annotations. Thus, the cilia-associated genes shared by these four ciliate species may represent evolutionarily stable, inherited cilia-associated genes in hypotrich ciliates.

Despite the commonality, the cilia-associated proteins of all four families belong to 50 different protein families, with the number and species of members varying among the different ciliates. This suggests that different ciliates contain different members of the cilia-associated gene families, which may have functional similarities and complementarity, enabling both evolutionary order and functional diversity among homologous proteins.

Previous studies have indicated significant functional variations of cilia-associated genes among different species, with notable structural and functional differences (Nevers et al., 2017; Kaku and Komatsu, 2017; Patir et al., 2020; Chen et al., 2023). Functional annotation of *E. amieti* genes indicated that a significant portion of the cilia-associated genes encode enzymes, with the top 20 proteins exhibiting enrichment in tubulin binding, microtubule motor activity, and dynein light intermediate chain binding. Functional classification of 223 cilia-associated proteins in *Tetrahymena thermophila* revealed that 14.3% of these proteins are motor proteins, the second largest protein group (Li et al., 2004). Given the crucial role of proteins with enzymatic or motor activities in cilia-associated assembly or disassembly (Liao et al., 2012), and the confirmation that nearly 60% of cilia-associated genes are required for cilia-associated differentiation or function in zebrafish (Li et al., 2019), it is reasonable to speculate that the majority of cilia-associated genes in ciliates are not structural genes but rather regulatory genes involved in cilia assembly or disassembly.

Localization annotation revealed the enrichment of the top 20 genes in the ciliary plasma, axoneme, and basal body. Consistent with this, data from *Chlamydomonas* genomes have demonstrated that a large percentage of known cilia-associated proteins are localized in the basal body or centrosome (Terán et al., 2021). Experimental investigations have further validated that approximately 20% of the cilia-associated proteins randomly selected from the ciliome exhibit localization in cilia or ciliated bases (Seixas et al., 2010). In *Trypanosoma brucei*, 60 cilia-associated proteins were found to localize at the membrane tips of axons and cilia, playing roles in sensing the surrounding environment, signaling, and motility (Mitchison and Valente, 2017). These findings suggest that the basal body is a significant site for the localization of cilia-associated proteins. A recent study on the interactome of cilia and centrosomes has elucidated the crucial involvement of centrosome proteins in connecting with cilia and various biological processes, including signaling pathway transduction, mitosis, and protein synthesis. Therefore, it is hypothesized that the cilia-associated proteins located in basal bodies in *E. amieti* may play a role in the regulation of ciliogenesis.

It was found that most cilia-associated genes in *E. amieti* do not contain introns, and some genes undergo programmed ribosomal frameshifting, indicating a unique gene structure. Programmed translation frameshifts occur when specific sequences in the mRNA manipulate the ribosome, causing it to change its reading frame (Pavel et al., 2002). There is a high level of programmed frameshifting in cilia-associated genes within the ciliary basal body, including BBS family proteins, CEP290, and δ-/ε- tubulin, which are also closely associated with ciliopathies (Chen et al., 2006). Given the high incidence of PRF in *Euplotes* and its important role in protein expression regulation (Wang et al., 2021), it is plausible that programmed ribosomal frameshifting represents a novel mechanism for regulating ciliary assembly and function in ciliates. The involvement of η-tubulin in basal body assembly as a programmed ribosomal frameshifting gene supports this notion (Liu et al., 2021). Furthermore, tubulin’s significant role in ciliary formation, along with the impact of primary cilia-associated gene mutations on skeletal, dental, and skin aspects, is noted. Therefore, it is proposed that ciliary proteins situated at the ciliary basal body are integral to crucial regulatory mechanisms governing ciliary assembly, and their protein expression is also precisely regulated at the translational level.

The expression and localization of different genes vary throughout different cell cycles. For instance, Cyclin D1 is expressed in the precursors of multiciliated cells and is downregulated during differentiation. CDK1 is localized in the nucleus of dividing multiciliated cells and moves to the cytoplasm in mature, quiescent multiciliated cells. The activity of CDK1 is downregulated during the division cycle, interfering with cell dynamics. Additionally, some inhibitory genes, such as *Cdkn1a*, are upregulated, further inhibiting the expression of CDK1. The dynamic expression and localization of these cell cycle regulatory factors suggest that they may coordinate the stages of multiciliated cell differentiation (Choksi et al., 2024). The formation of cilia and cell division are mutually exclusive processes. Cilia are shed twice during the cell cycle: once before entering the S phase and again between the S phase and M phase (Sehyun and Leonidas, 2011). In *Chlamydomonas*, ciliary loss occurs through two mechanisms: resorption and shedding. The ciliary loss before cell division is due to resorption, which involves the precise severing of the nine outer doublet microtubules at the ciliary base, occurring at the distal transition zone between the basal body and the flagellum, known as the site of flagellar autotomy (SOFA) (Hildebrandt et al., 2009). RT-PCR has shown that a third of the 103 genes examined were transcriptionally upregulated 3- to 13-fold during the process of *Chlamydomonas* flagellar regeneration (Liao et al., 2012). Another study demonstrated that 41% of predicted genes were expressed during flagellar regeneration using maskless photolithographic DNA synthesis technology, with 33 out of 61 known flagellar genes exhibiting RNA expression levels that were 175% or higher (Li et al., 2019). Conversely, the current study revealed that only 6 out of 48 genes were upregulated during morphogenesis.

It is important to note that the timing of cell examination differed between studies. This study conducted assessments 8 h after feeding, while the previous study examined *Chlamydomonas* at 30, 45, or 120 min after deflagellation. This discrepancy in time points may have influenced the expression levels of various genes. Additionally, the genes examined in this study differ from those in previous studies, and different genes of the same gene family may play varied roles in the same biological process. In summary, it is speculated that ciliary resorption and depolymerization occur in *E. amieti* during the division phase, with the main cellular activity being cell division.

Most cilia-associated genes have been confirmed to be linked with ciliopathies (Wallmeier et al., 2020; Hildebrandt et al., 2009; Karalis et al., 2022; Handa et al., 2020; Chen et al., 2021). In this study, five hub genes were identified as being highly correlated with ciliopathies, suggesting that these genes play critical roles in ciliogenesis and could serve as targets for future research into the molecular mechanisms underlying these diseases. This research provides a more detailed examination of cilia-associated genes in *E. amieti*, focusing particularly on their potential connection to ciliopathies. The RNAi experiments on two randomly selected ciliary genes confirmed their influence on cilia dynamics.

*DYNLRB2* (Dynein Light Chain Roadblock-Type 2) is essential for cilia assembly and function. As a component of the dynein complex, DYNLRB2 assists in transporting proteins and other molecules necessary for cilia construction and provides structural support to ensure cilia stability and functionality. During cilia assembly, microtubules extend from the basal body to form the axoneme, with DYNLRB2 interacting with other dynein subunits to drive microtubule sliding, promoting cilia extension and movement. Deficiency in DYNLRB2 can lead to ciliary dysfunction, affecting normal cellular activities and functions (Toropova et al., 2019; He et al., 2023). *DYNLRB2* is also important in male meiosis and reproduction, with gene expression upregulated in the testis, particularly in meiotic cells (He et al., 2023). Consequently, a defect in DYNLRB2 can result in reduced sperm motility and infertility (Terán et al., 2021). Additionally, *DYNLRB2* plays a role in brain development and its deficiency is linked to Alzheimer’s disease (Xia et al., 2022). Mutations in DYNLRB2 have been associated with intraductal carcinoma of the breast, hypercholesterolemia, Jeune syndrome, short rib polydactyly type III, asphyxiating thoracic dystrophy, and abnormal cranial development in embryonic infants (Liao et al., 2012; Li et al., 2019; Pina et al., 2023; Asante et al., 2014).

*ARL2BP* is critical for various cellular functions. Studies have reported the presence of ARL2BP protein in the axonemes and basal bodies of cilia-related cells (Cai et al., 2019). In cultured cells, ARL2BP specifically localizes to the centrosomes and midbodies of actively dividing cells. In the mouse retina, ARL2BP is found in the basal bodies and cilia-associated centrioles of photoreceptor cells, as well as in the ciliary extension of the inner segment. While primarily located in the cytoplasm, ARL2BP has also been detected in mitochondria, where it forms a complex with the adenine nucleotide transporter protein (ANT) (Sharer et al., 2002). The *ARL2BP* gene encodes the ARL2 protein, which is an effector protein of ARL2 and ARL3, localized to the distal connecting cilia of photoreceptors and the basal bodies of cilia-related cells. Loss of ARL2BP leads to cilia shortening (Davidson et al., 2013). ARL2BP is linked to retinitis pigmentosa and is essential for the maintenance and function of normal photoreceptors (Davidson et al., 2013). It is located at the connecting cilia of photoreceptor cells and is necessary for forming ciliary doublets and axoneme elongation (Moye et al., 2018). Deficiency in *ARL2BP* results in abnormal doublet microtubule structures in the axoneme. Homozygous mutations in ARL2BP can lead to autosomal recessive retinitis pigmentosa (Cai et al., 2019; Davidson et al., 2013). In three unrelated families, variants of ARL2BP have been reported as potential causes of retinitis pigmentosa and may also be associated with organ laterality defects. Mutations in ARL2BP can cause recessive rod-cone dystrophies (Audo et al., 2017). *ARL2BP* is vital for maintaining cilia structure and sperm flagella, with its deficiency leading to syndromic ciliopathies (Moye et al., 2019).

## Conclusion

In this study, we sequenced the entire genome of *E. amieti*, utilized online analysis software and existing gene libraries for a comprehensive genomic analysis, and identified 418 cilia-associated genes. RT-PCR analysis revealed that the expression of some cilia-associated proteins was significantly downregulated during the cell division period. To further investigate the functional roles of these genes, two were randomly selected for gene interference experiments, which resulted in alterations to the cytoskeleton and disruptions in the swimming and survival patterns of *E. amieti*. Our findings indicate that the ciliary structure of ciliates is highly susceptible to changes due to gene loss, particularly during interphase, when critical cellular activities occur. The identified cilia-associated genes also show significant homology with human genes, suggesting that ciliates can serve as valuable pathological models for genetic research into ciliopathies. This study provides foundational insights that can be used to explore the molecular mechanisms of ciliopathies and develop potential therapeutic strategies.

## Data Availability

The datasets presented in this study can be found in online repositories. The names of the repository/repositories and accession number(s) can be found in the article/Supplementary material.

## Footnotes

1
http://cildb.i2bc.paris-saclay.fr

2
https://evan.ciliate.org/index.php/home/index.php

3
http://ciliates.ihb.ac.cn/database/home/#eo

4
https://oxy.ciliate.org/index.php/home/welcome

5
https://stylo.ciliate.org/index.php/home/index.php

6
https://www.ncbi.nlm.nih.gov/refseq/about/non-redundantproteins/

7
http://www.webgestalt.org

8
http://metascape.org/

9
https://www.geneontology.org

10
https://meme-suite.org/meme/

11
http://pfam.xfam.org/

12
https://cn.string-db.org/

13
https://cytoscape.org/
