# Supplementary material for: Identification and bioinformatics analysis of cilia-associated gene families in Euplotes amieti (Ciliophora, Hypotrichia)
Source: Front Microbiol. 2025 May 13;16:1486189. doi: 10.3389/fmicb.2025.1486189 (PMC12106347; doi:10.3389/fmicb.2025.1486189)
Supplement: Supplementary file 1 [file Table_1.docx]

| ***E. amieti*** | ***E.octocarinatus*** | ***S. lemnae*** | ***O. trifallax*** | **44 genes common with all the four spacies** | **12 genes common with three, but not in O. trifallax** | **25 genes common with the three, but not in E. amieti** | **10 genes common with the three, but not in E. octocarinatus** | **6 genes common in the three, but not in S. lemnae** |
| --- | --- | --- | --- | --- | --- | --- | --- | --- |
| ABP1 | ABCB7 | ABCB8 | ADCY3 | alpha tubulin | Casein kinase I isoform delta | AGD5 | ARL2 | ADCY3 |
| ACAP2 | ABCC11 | AGD5 | AGD5 | ARF1 | centromere protein j | AGD9 | ATAT1 | fgfr1 oncogene partner |
| actin II | ADCY3 | AGD9 | AGD9 | ARL6 | CEP41 | ARF | BBS2 | RAB10 |
| ACTL1 | AGD5 | alpha tubulin | alpha tubulin | B9D1 | DNM2 | B9D2 | DNAAF3 | RAB2B |
| ACTR1A | AGD9 | ARF | ARF | BBS1 | gamma-tubulin complex | Casein kinase I isoform alpha | IFT172 | thioredoxin |
| ADCY3 | alpha tubulin | ARF1 | ARF1 | BBS4 | iq domain-containing protein d | Casein kinase II subunit beta | KIF3 | Tectnic-1 |
| ADGB | ARF | ARFGAP1 | ARF2b | BBS5 | KIF15 | CCDC94 | LRRC51 |  |
| ADH5 | ARF1 | ARL1 | ARF5 | beta tubulin | myosin head | ctxp80 | RSPH |  |
| Advillin | ARFGAP1 | ARL14 | ARI8 | CEP104 | PKD2L1 | DNAL1 | tubulin specific chaperone d |  |
| AGC kinase | ARL11 | ARL2 | ARL | CEP135 | rab gdp dissociation inhibitor | IFT20 | tubulin tyrosine ligase like 12 |  |
| AK4 | ARL4C | ARL6 | ARL1 | delta Tubulin | RAB28 | iq calmodulin-binding motif family protein |  |  |
| AK7 | ARL6 | ARL8B | ARL2 | DNAH1 | TTC27 | LRRC61 |  |  |
| AKT2 | arrestin | ATAT1 | ARL3 | DNAI2 |  | LRRC9 |  |  |
| alpha tubulin | ATP-binding cassette | ATG16 | ARL6 | DNM1 |  | MYH7 |  |  |
| ANK3 | Axoneme central apparatus protein | ATG3 | ARRDC1 | DYNC2LI1 |  | NEK |  |  |
| ARF1 | B9D1 | ATG5 | ATAT1 | DYNLL1 |  | NEK2 |  |  |
| ARFGEF1 | B9D2 | ATG8E | B9D1 | DYNLRB2 |  | serine threonine-protein kinase prp4 |  |  |
| ARI | BBS1 | ATG8F | B9D2 | EB1 |  | serine threonine-protein phosphatase |  |  |
| ARL | BBS4 | atp binding microtubule motor family isoform 1 | BBS1 | gamma-tubulin |  | serine threonine-protein phosphatase 2a regulatory subunit b subunit gamma |  |  |
| ARL2 | BBS5 | axonemal dynein light chain p33 | BBS2 | IFT122 |  | serine threonine-protein phosphatase dullard |  |  |
| ARL2BP | beta tubulin | axoneme central apparatus protein | BBS4 | IFT27 |  | TTC |  |  |
| ARL3 | beta tubulin cofactor a | B9D1 | BBS5 | IFT46 |  | tubby-related protein 1 |  |  |
| ARL6 | casein kinase | B9D2 | BBS7 | IFT52 |  | tubulin tyrosine ligase family |  |  |
| ARRDC3 | casein kinase I | basal body protein | beta tubulin | IFT74 |  | tubulin tyrosine ligase like 5 |  |  |
| arrestin | Casein kinase I isoform delta | BBS1 | Casein kinase | IFT80 |  | ubiquitin-activating enzyme e1 family protein |  |  |
| ATAD3A | Casein kinase I isoform alpha | BBS2 | Casein kinase I | IFT81 |  |  |  |  |
| ATAT1 | Casein kinase II subunit beta | BBS4 | Casein kinase I isoform alpha | IFT88 |  |  |  |  |
| ATG1 | CCDC25 | BBS5 | Casein kinase I isoform delta | LRRC |  |  |  |  |
| ATG3 | CCDC94 | BBS7 | Casein kinase I isoform epsilon | LRRC23 |  |  |  |  |
| ATG5 | Central apparatus associated protein | beta tubulin | Casein kinase II subunit alpha | LRRC40 |  |  |  |  |
| ATG8 | central pair associated wd repeat protein | beta-tubulin folding cofactor a | Casein kinase II subunit beta | LRRC48 |  |  |  |  |
| ATP8A2 | centromere microtubule binding protein | casein kinase | CCDC113 | LRRC56 |  |  |  |  |
| ATR | centromere protein j | Casein kinase I isoform delta | CCDC13 | nephrocystin-4 |  |  |  |  |
| B9D1 | CEP104 | casein kinase II | CCDC153 | POC1A |  |  |  |  |
| BBS1 | CEP135 | Casein kinase I | CCDC22 | Polycystin 2 |  |  |  |  |
| BBS2 | CEP41 | Casein kinase I isoform alpha | CCDC25 | RSPH1 |  |  |  |  |
| BBS4 | ctxp80 | Casein kinase II subunit alpha | CCDC2A | RSPH3 |  |  |  |  |
| BBS5 | Cytoplasmic dynein light intermediate chain | Casein kinase II subunit beta | CCDC39 | serine threonine-protein phosphatase 5 |  |  |  |  |
| bbs7 | delta Tubulin | CCDC104 | CCDC40 | TMEM231 |  |  |  |  |
| BBS9 | DH1C | CCDC108 | CCDC42 | TTC26 |  |  |  |  |
| beta tubulin | DNAH1 | CCDC94 | CCDC65 | TTC4 |  |  |  |  |
| betaine aldehyde dehydrogenase | DNAH10 | central pair associated wd repeat protein | CCDC93 | TTC5 |  |  |  |  |
| BRD4 | DNAH6 | centromere associated protein e | CCDC94 | TTLL1 |  |  |  |  |
| C11orf49 | DNAI2 | centromere microtubule binding protein | Central apparatus associated protein | Ubiquitin-activating enzyme |  |  |  |  |
| CAF1 | DNAL1 | centromere protein j | centromere protein j |  |  |  |  |  |
| Calcineurin subunit B | DNAL10 | CEP104 | Centrosomal protein of isoform B |  |  |  |  |  |
| Calcium-dependent protein kinase | DNAL28A | CEP135 | CEP104 |  |  |  |  |  |
| Calcium-transporting ATPase 1 | DNAL28B | CEP41 | CEP135 |  |  |  |  |  |
| calcyphosin-like protein | DNAL6 | CEP44 | CEP44 |  |  |  |  |  |
| CaM | DNM1 | CEP97 | CEP78 |  |  |  |  |  |
| Casein kinase I isoform alpha | DNM2 | ctxp80 | CEP97 |  |  |  |  |  |
| Casein kinase I isoform delta | dynamin central region family protein | cytoskeleton-associated protein 5 | CFAP206 |  |  |  |  |  |
| casein kinase II subunit alpha | DYNC1I2 | delta Tubulin | CFAP45 |  |  |  |  |  |
| Casein kinase II subunit beta | DYNC2LI1 | DNAAF3 | CFAP52 |  |  |  |  |  |
| CCDC108 | DYNLL1 | DNAH1 | CFAP58 |  |  |  |  |  |
| CCDC113 | DYNLRB2 | DNAI1 | CLUAP1 |  |  |  |  |  |
| CCDC13 | e3 ubiquitin-protein ligase | DNAI2 | Component of dynein regulatory complex |  |  |  |  |  |
| CCDC146 | e3 ubiquitin-protein ligase ATL4 | DNAL1 | crystallin |  |  |  |  |  |
| CCDC151 | e3 ubiquitin-protein ligase BRE1B | DNAL28a | CSNK2A1 |  |  |  |  |  |
| CCDC176 | e3 ubiquitin-protein ligase CHIP | DNM1 | ctxp80 |  |  |  |  |  |
| CCDC22 | e3 ubiquitin-protein ligase HECTD1 | DNM2 | Cullin |  |  |  |  |  |
| CCDC2A | e3 ubiquitin-protein ligase HERC2 | dynamin central region family protein | Cytoskeleton-associated protein 5 |  |  |  |  |  |
| CCDC39 | e3 ubiquitin-protein ligase HERC4 | DYNC1H1 | DAW1 |  |  |  |  |  |
| CCDC40 | e3 ubiquitin-protein ligase LUL3 | DYNC1LI2 | delta Tubulin |  |  |  |  |  |
| CCDC42 | e3 ubiquitin-protein ligase MARCH2 | DYNC2LI1 | DLC90F |  |  |  |  |  |
| CCDC63 | e3 ubiquitin-protein ligase nrdp1 | dynein cytoplasmic intermediate polypeptide 2 | DNAAF3 |  |  |  |  |  |
| CCDC65 | e3 ubiquitin-protein ligase Os04g0590900 | DYNLL1 | DNAH1 |  |  |  |  |  |
| CCDC93 | e3 ubiquitin-protein ligase RBX1 | DYNLRB1 | DNAH4 |  |  |  |  |  |
| CDC48 | e3 ubiquitin-protein ligase RING1 | DYNLRB2 | DNAH7 |  |  |  |  |  |
| CDKL5 | e3 ubiquitin-protein ligase RNF126 | e3 ubiquitin-protein ligase nrdp1 | DNAI2 |  |  |  |  |  |
| centrin | e3 ubiquitin-protein ligase RNF14 | e3 ubiquitin-protein ligase ring1 | DNAL1 |  |  |  |  |  |
| centromere associated protein e | e3 ubiquitin-protein ligase RNF170 | e3 ubiquitin-protein ligase rkp | DNALI1 |  |  |  |  |  |
| centromere protein j | e3 ubiquitin-protein ligase RNF6 | e3 ubiquitin-protein ligase RNF115 | DNM1 |  |  |  |  |  |
| CEP104 | e3 ubiquitin-protein ligase RNF8 | e3 ubiquitin-protein ligase RNF13 | DYNC2I1 |  |  |  |  |  |
| CEP120 | e3 ubiquitin-protein ligase SDIR1 | e3 ubiquitin-protein ligase RNF14 | DYNC2LI1 |  |  |  |  |  |
| CEP131 | e3 ubiquitin-protein ligase TRIM21 | e3 ubiquitin-protein ligase RNF144a | Dynein 18 kDa light chain, flagellar outer arm |  |  |  |  |  |
| CEP135 | e3 ubiquitin-protein ligase TRIM37 | e3 ubiquitin-protein ligase RNF149 | Dynein cytoplasmic intermediate polypeptide 2 |  |  |  |  |  |
| CEP162 | e3 ubiquitin-protein ligase TTC3 | e3 ubiquitin-protein ligase RNF25 | dynein light intermediate chain |  |  |  |  |  |
| CEP19 | EB1 | e3 ubiquitin-protein ligase sdir1 | Dynein outer arm light chain |  |  |  |  |  |
| CEP290 | fgfr1 oncogene partner | e3 ubiquitin-protein ligase sis3 | Dynein-1-alpha heavy chain |  |  |  |  |  |
| CEP41 | flagellar associated protein | e3 ubiquitin-protein ligase TRIM23 | DYNLL1 |  |  |  |  |  |
| CEP76 | flagellar basal body protein | e3 ubiquitin-protein ligase TRIM37 | DYNLRB2 |  |  |  |  |  |
| CFAP 69 | flagellar radial spoke protein | e3 ubiquitin-protein ligase ubr4 | e3 ubiquitin-protein ligase |  |  |  |  |  |
| CFAP119 | gamma-tubulin | e3 ubiquitin-protein ligase zswim2 | e3 ubiquitin-protein ligase ARI8 |  |  |  |  |  |
| CFAP206 | gamma-tubulin complex | EB1 | e3 ubiquitin-protein ligase Arkadia |  |  |  |  |  |
| CFAP221 | GNB2L1 | EB2 | e3 ubiquitin-protein ligase BRE1B |  |  |  |  |  |
| CFAP251 | GNL3L | EML6 | e3 ubiquitin-protein ligase CHFR |  |  |  |  |  |
| CFAP299 | hect e3 ubiquitin | fantom | e3 ubiquitin-protein ligase chip |  |  |  |  |  |
| CFAP300 | IFT122 | flagellar associated protein | e3 ubiquitin-protein ligase HECTD1 |  |  |  |  |  |
| CFAP36 | IFT20 | gamma-tubulin | e3 ubiquitin-protein ligase HERC2 |  |  |  |  |  |
| CFAP45 | IFT27 | gamma-tubulin complex | e3 ubiquitin-protein ligase HERC4 |  |  |  |  |  |
| CFAP46 | IFT46 | GNB2L1 | e3 ubiquitin-protein ligase MARCH6 |  |  |  |  |  |
| CFAP52 | IFT52 | IFT122 | e3 ubiquitin-protein ligase nrdp1 |  |  |  |  |  |
| CFAP58 | IFT74 | IFT140 | e3 ubiquitin-protein ligase ring1 |  |  |  |  |  |
| CFAP61 | IFT80 | IFT172 | e3 ubiquitin-protein ligase RNF13 |  |  |  |  |  |
| CFAP70 | IFT81 | IFT20 | e3 ubiquitin-protein ligase RNF149 |  |  |  |  |  |
| CFAP74 | IFT88 | IFT27 | e3 ubiquitin-protein ligase RNF25 |  |  |  |  |  |
| CFAP77 | iq calmodulin-binding motif family protein | IFT46 | e3 ubiquitin-protein ligase RNF6 |  |  |  |  |  |
| CFAP91 | iq domain-containing protein d | IFT52 | e3 ubiquitin-protein ligase rpm1 |  |  |  |  |  |
| Chaperonin beta subunit | Jouberin | IFT74 | e3 ubiquitin-protein ligase synoviolin |  |  |  |  |  |
| chaperonin containing TCP1 | KIF | IFT80 | e3 ubiquitin-protein ligase TRIM37 |  |  |  |  |  |
| Clathrin heavy chain | KIF14 | IFT81 | EB1 |  |  |  |  |  |
| CLUAP1 | KIF15 | IFT88 | EML6 |  |  |  |  |  |
| CNBD2 | KIF2A | iq calmodulin-binding motif family protein | fgfr1 oncogene partner |  |  |  |  |  |
| cofilin | KIF7 | iq domain-containing protein d | flagellar associated protein |  |  |  |  |  |
| COPS8 | LRRC | Jouberin | Flagellar outer dynein arm intermediate chain 1 |  |  |  |  |  |
| CREB3L1 | LRRC23 | KIF | Flagellar outer dynein arm-docking complex |  |  |  |  |  |
| CSNK2B | LRRC40 | KIF15 | flagellar basal body protein |  |  |  |  |  |
| CTR1 | LRRC46 | KIF2 | flagellar radial spoke protein |  |  |  |  |  |
| ctr1 | LRRC48 | KIF3 | gamma-tubulin |  |  |  |  |  |
| CYB5D1 | LRRC56 | KIF4 | gamma-tubulin complex |  |  |  |  |  |
| cysteinyl-trna synthetase | LRRC61 | KIF4A | Guanine nucleotide-binding protein subunit beta-1 |  |  |  |  |  |
| cytoskeleton-associated protein 5 | LRRC9 | kinesin heavy chain | GYP1 |  |  |  |  |  |
| delta Tubulin | LRRIQ3 | kinesin light chain | hect e3 ubiquitin |  |  |  |  |  |
| DHC1 | MAP | Kinesin like protein | IFT |  |  |  |  |  |
| dihydrolipoyl dehydrogenase | microtubule binding protein | Kinesin-domain-containing protein | IFT122 |  |  |  |  |  |
| DNAAF1 | MKS4 | klp1 | IFT172 |  |  |  |  |  |
| DNAAF3 | MYH7 | leucine-rich repeat-containing protein loc400891-like | IFT20 |  |  |  |  |  |
| DNAAF6 | myosin head | Leucine-rich repeat-containing protein typical subtype | IFT27 |  |  |  |  |  |
| DNAH1 | Myosin heavy chain | LRRC | IFT46 |  |  |  |  |  |
| DNAH7 | NEK | LRRC23 | IFT52 |  |  |  |  |  |
| DNAI2 | NEK2 | LRRC34 | IFT74 |  |  |  |  |  |
| DNAJB13 | nephrocystin-4 | LRRC40 | IFT80 |  |  |  |  |  |
| DNAJB6 | PKD2 | LRRC43 | IFT81 |  |  |  |  |  |
| DNAL28a | PKD2L1 | LRRC48 | IFT88 |  |  |  |  |  |
| DNM1 | POC1A | LRRC51 | iq calmodulin-binding motif family protein |  |  |  |  |  |
| DNM1L | Polycystin 2 | LRRC56 | jouberin |  |  |  |  |  |
| DNM2 | rab gdp dissociation inhibitor | LRRC61 | KIF1 |  |  |  |  |  |
| DPCD | rab gtpase-activating protein 1-isoform 10-like | LRRC9 | KIF16 |  |  |  |  |  |
| DRC1 | RAB10 | LRRCC1 | KIF3 |  |  |  |  |  |
| DRC7 | RAB11 | LRRIQ3 | KIF5 |  |  |  |  |  |
| Dynactin | RAB11a | meckelin | kinesin light chain |  |  |  |  |  |
| DYNC1H1 | RAB11b | microtubule-associated protein spiral2-like | Kinesin like protein |  |  |  |  |  |
| DYNC1I2 | RAB13 | MKS4 | Kinesin heavy chain |  |  |  |  |  |
| DYNC2H1 | RAB14 | MYH7 | Kinesin motor catalytic domain protein |  |  |  |  |  |
| DYNC2LI1 | RAB18 | myosin | Kinesin motor protein |  |  |  |  |  |
| Dynein light chain 1 | RAB1A | myosin head | Kinesin-domain-containing protein |  |  |  |  |  |
| dynein light chain 2 | RAB2 | myosin heavy chain | KRP85 |  |  |  |  |  |
| DYNLL1 | RAB26 | myosin light chain | Leucine-rich repeat-containing protein typical subtype |  |  |  |  |  |
| DYNLRB2 | RAB28 | NEK | LRRC |  |  |  |  |  |
| E1-like protein-activating | RAB2a | NEK10 | LRRC23 |  |  |  |  |  |
| E3 ubiquitin-protein ligase CHFR | RAB2B | NEK2 | LRRC34 |  |  |  |  |  |
| E3 ubiquitin-protein ligase HERC2 | RAB2C | NEK3 | LRRC40 |  |  |  |  |  |
| E3 ubiquitin-protein ligase RNF6 | RAB2D | NEK5 | LRRC43 |  |  |  |  |  |
| E3 ubiquitin-protein ligase TRIM37 | RAB30 | nephrocystin-4 | LRRC46 |  |  |  |  |  |
| EB1 | RAB32 | outer row dynein assembly protein 16 homolog | LRRC48 |  |  |  |  |  |
| EF hand family protein | RAB37 | PKD2L1 | LRRC51 |  |  |  |  |  |
| EFCAB1 | RAB3D | POC1A | LRRC56 |  |  |  |  |  |
| efhc2 | RAB43 | Polycystin 2 | LRRC61 |  |  |  |  |  |
| eh domain containing protein | RAB5 | rab gdp dissociation inhibitor | LRRC9 |  |  |  |  |  |
| eIF4A3 | Rab5 GDP/GTP exchange factor | RAB27A | LRRCC1 |  |  |  |  |  |
| ELMO domain-containing protein 3-like | RAB5A | RAB28 | Meckelin |  |  |  |  |  |
| elongation factor 2 | Rab5-interacting protein | RAb30 | Microtubule-binding protein |  |  |  |  |  |
| EML5 | RAB6 | RAb32 | MORN40 |  |  |  |  |  |
| Enkurin | RAB7 | RAB35 | MYH7 |  |  |  |  |  |
| Enkurin domain-containing protein 1 | RAB7a | RAB39B | myosin heavy chain |  |  |  |  |  |
| ERDJ2 | RAB8 | RAb3C | myosin light chain |  |  |  |  |  |
| EVI5 | RAB8A | RAb4 | NEK |  |  |  |  |  |
| extracellular response kinase | RABA2a | RAB4B | NEK1 |  |  |  |  |  |
| FAM161B | RABA5a | RAB5A | NEK10 |  |  |  |  |  |
| fantom | RABA61 | RAb7 | NEK2 |  |  |  |  |  |
| fgfr1 oncogene partner | RABEPK | RAb7A | NEK4 |  |  |  |  |  |
| fibrocystin-L | RabGAP/TBC | RAb8B | nephrocystin-4 |  |  |  |  |  |
| Flagellar outer dynein arm light chain 2 | RABGAP1L | radial spoke head containing protein | ODAD3 |  |  |  |  |  |
| GABARAP | RabX27 | RSPH | Outer dynein arm docking complex protein oda-dc |  |  |  |  |  |
| gamma-tubulin | RabX33 | RSPH1 | PKD2 |  |  |  |  |  |
| gamma-tubulin complex | Radial spoke head-L protein | RSPH10 | POC1A |  |  |  |  |  |
| gas2 | RING-finger-containing E3 ubiquitin ligase | RSPH3 | POC5 |  |  |  |  |  |
| GAS8 | RSPH1 | RSPH7 | Polycystin 2 |  |  |  |  |  |
| Glucose-6-phosphate isomerase | RSPH3 | serine threonine-protein kinase mak | Protein serine threonine kinase |  |  |  |  |  |
| glycogen synthase kinase-3 beta | serine threonine protein kinase | serine threonine-protein kinase osr1 | Rab family gtpase |  |  |  |  |  |
| GNB2 | serine threonine-protein kinase atr | serine threonine-protein kinase partial | RAB GTPase |  |  |  |  |  |
| GNL3L | serine threonine-protein kinase osr1 | serine threonine-protein kinase pk61c | Rab small monomeric gtpase |  |  |  |  |  |
| GSP1 | serine threonine-protein kinase prp4 | serine threonine-protein kinase plk4 | rab subfamily protein of small gtpase |  |  |  |  |  |
| GST1 | serine threonine-protein kinase rio1 | serine threonine-protein kinase prp4 | RAB10 |  |  |  |  |  |
| GTPBP4 | serine threonine-protein phosphatase | serine threonine-protein kinase rio1 | RAB19 |  |  |  |  |  |
| heat repeat-containing protein 2 | serine threonine-protein phosphatase 2a 65 kda regulatory subunit a alpha | serine threonine-protein kinase ulk2 | RAB23 |  |  |  |  |  |
| hect e3 ubiquitin | serine threonine-protein phosphatase 2a 65 kda regulatory subunit a beta | serine threonine-protein kinase ulk4 | RAB2B |  |  |  |  |  |
| high mobility group protein B2 | serine threonine-protein phosphatase 2a regulatory subunit b | serine threonine-protein phosphatase | RAB2C |  |  |  |  |  |
| histone deacetylase 6 | serine threonine-protein phosphatase 2a regulatory subunit b subunit gamma | serine threonine-protein phosphatase 2a 55 kda regulatory subunit b beta isoform | RAB5 |  |  |  |  |  |
| HOP | serine threonine-protein phosphatase 5 | serine threonine-protein phosphatase 2a 65 kda regulatory subunit a alpha isoform-like | Rab5 GDP/GTP exchange factor |  |  |  |  |  |
| HSP70 | serine threonine-protein phosphatase dullard | serine threonine-protein phosphatase 2a 65 kda regulatory subunit a beta isoform-like | Rab5A |  |  |  |  |  |
| HSP90 | sperm flagellar protein 2 | serine threonine-protein phosphatase 2a catalytic subunit alpha isoform | RAB7 |  |  |  |  |  |
| hydrocephalus-inducing protein | SRK2C | serine threonine-protein phosphatase 2a regulatory subunit b | RAB7A |  |  |  |  |  |
| IFT122 | Tau-tubulin kinase 2 | serine threonine-protein phosphatase 2a regulatory subunit b subunit alpha-like | RABEPK |  |  |  |  |  |
| IFT140 | Tectonic-1 | serine threonine-protein phosphatase 2a regulatory subunit b subunit gamma | RABF1 |  |  |  |  |  |
| IFT172 | Tectonic-3 | serine threonine-protein phosphatase 4 catalytic subunit | RABGGTB |  |  |  |  |  |
| IFT22 | tetraspanin family protein | serine threonine-protein phosphatase 4 regulatory subunit 4 | Rab-like gtpase activating |  |  |  |  |  |
| IFT27 | thioredoxin | serine threonine-protein phosphatase 5 | Rab-type small GTPase |  |  |  |  |  |
| IFT46 | TMEM231 | serine threonine-protein phosphatase 6 regulatory ankyrin repeat subunit a-like | Rac-beta serine threonine-protein kinase |  |  |  |  |  |
| IFT52 | tortifolia1-like | serine threonine-protein phosphatase 6 regulatory ankyrin repeat subunit b-like | Radial spoke head-L protein |  |  |  |  |  |
| IFT57 | transmembrane protein | serine threonine-protein phosphatase dullard | RING-finger-containing E3 ubiquitin ligase |  |  |  |  |  |
| IFT74 | TTC | serine threonine-protein phosphatase pp1 | RSPH |  |  |  |  |  |
| IFT80 | TTC26 | sperm flagellar protein 2 | RSPH1 |  |  |  |  |  |
| IFT81 | TTC27 | Tau-tubulin kinase 1 | RSPH10 |  |  |  |  |  |
| IFT88 | TTC4 | Tau-tubulin kinase 2 | RSPH3 |  |  |  |  |  |
| importin-5 | TTC5 | tetraspanin family protein | RSPH7 |  |  |  |  |  |
| inner dynein arm light chain | TTLL1 | tetratricopeptide repeat domain containing protein | serine threonine protein kinase |  |  |  |  |  |
| iq and aaa domain-containing protein 1 | TTLL13 | tetratricopeptide tpr_1 repeat-containing protein | serine threonine protein kinase with wd40 repeats |  |  |  |  |  |
| iq and ubiquitin-like domain-containing | tubby protein | tetratricopeptide-like helical domain-containing protein | serine threonine protein phosphatase |  |  |  |  |  |
| iq domain-containing protein d | tubby-related protein 1 | thioredoxin dynein outer arm protein | serine threonine-protein kinase prp4 |  |  |  |  |  |
| jade-3 | tubulin alpha-1 chain | TMEM | serine threonine-protein phosphatase |  |  |  |  |  |
| jouberin | tubulin alpha-1B chain | TMEM136 | serine threonine-protein phosphatase 2a 55 kda regulatory subunit b beta isoform |  |  |  |  |  |
| katanin p60 atpase-containing subunit a1 | tubulin alpha-1C chain | TMEM165 | serine threonine-protein phosphatase 2a regulatory subunit b subunit gamma |  |  |  |  |  |
| KIF12B | tubulin alpha-2 chain | TMEM20 | serine threonine-protein phosphatase 4 regulatory subunit 4-like |  |  |  |  |  |
| KIF13A | tubulin beta-1 chain | TMEM231 | serine threonine-protein phosphatase 5 |  |  |  |  |  |
| KIF13B | tubulin binding cofactor c domain-containing protein | TMEM43 | serine threonine-protein phosphatase 6 regulatory ankyrin repeat subunit a-like |  |  |  |  |  |
| KIF15 | tubulin folding cofactor C | TMEM56 | serine threonine-protein phosphatase dullard |  |  |  |  |  |
| KIF15A | tubulin glycylase 3C | TMEM63C | small rab-related gtpase |  |  |  |  |  |
| KIF17 | tubulin subunit beta | TMEM87B | sperm flagellar protein 1 |  |  |  |  |  |
| KIF19 | tubulin subunit TubB | TMEM93 | sporangia induced sperm flagellar protein |  |  |  |  |  |
| KIF24 | tubulin tyrosine ligase family | TOR1L1 | Tau-tubulin kinase 2 |  |  |  |  |  |
| KIF28P | tubulin tyrosine ligase like 5 | transient receptor potential cation subfamily member 4 | TBC domain-containing protein |  |  |  |  |  |
| KIF2A | tubulin, beta 4A class IVa | transmembrane protein | Tectonic-1 |  |  |  |  |  |
| KIF3 | tubulin/FtsZ family, GTPase domain protein | trp protein for ciliary function | thioredoxin |  |  |  |  |  |
| KIF3B | ubiquitin-activating enzyme e1 family protein | TTC | TMEM |  |  |  |  |  |
| KIF3C | Ubiquitin-activating enzyme | TTC1 | TMEM136 |  |  |  |  |  |
| KIF4A | ypt1 | TTC16 | TMEM165 |  |  |  |  |  |
| KIF5 |  | TTC21B | TMEM20 |  |  |  |  |  |
| KIF6 |  | TTC26 | TMEM222 |  |  |  |  |  |
| KIFAP3 |  | TTC27 | TMEM231 |  |  |  |  |  |
| KIFC1 |  | TTC30A | TMEM43 |  |  |  |  |  |
| klp1 |  | TTC4 | TMEM56 |  |  |  |  |  |
| lish domain-containing protein |  | TTC5 | TMEM63c |  |  |  |  |  |
| long flagella protein lf4 |  | TTLL1 | TMEM87b |  |  |  |  |  |
| LRGUK |  | TTLL2 | Transient receptor potential cation subfamily member 4 |  |  |  |  |  |
| LRRC |  | TTLL6 | Transmembrane protein |  |  |  |  |  |
| LRRC23 |  | tubby-related protein 1 | TTC |  |  |  |  |  |
| LRRC40 |  | tubulin binding cofactor c domain-containing protein | TTC1 |  |  |  |  |  |
| LRRC48 |  | tubulin epsilon chain | TTC16 |  |  |  |  |  |
| LRRC51 |  | tubulin folding cofactor | TTC21b |  |  |  |  |  |
| LRRC56 |  | tubulin polyglutamylase complex | TTC26 |  |  |  |  |  |
| Malate dehydrogenase 1 |  | tubulin specific chaperone d | TTC4 |  |  |  |  |  |
| MAPK1 |  | tubulin tyrosine ligase family | TTC5 |  |  |  |  |  |
| MARK3 |  | tubulin tyrosine ligase like 12 | TTLL1 |  |  |  |  |  |
| microtubule-associated protein |  | tubulin tyrosine ligase like 5 | TTLL6 |  |  |  |  |  |
| mitotic specific cyclin |  | ubiquitin-activating enzyme e1 family protein | tubby-related protein 1 |  |  |  |  |  |
| MKS3 |  | Ubiquitin-activating enzyme | tubulin folding cofactor B like |  |  |  |  |  |
| MNS1 |  |  | tubulin specific chaperone d |  |  |  |  |  |
| morn repeat protein |  |  | tubulin tyrosine ligase family |  |  |  |  |  |
| MORN2 |  |  | tubulin tyrosine ligase like 12 |  |  |  |  |  |
| MORN5 |  |  | tubulin tyrosine ligase like 5 |  |  |  |  |  |
| multidrug resistance-associated protein 1 |  |  | tubulin binding cofactor c domain-containing protein |  |  |  |  |  |
| MYG1 |  |  | tubulin epsilon chain |  |  |  |  |  |
| MYH1 |  |  | tubulin folding cofactor |  |  |  |  |  |
| MYH10 |  |  | tubulin glycylase 3D |  |  |  |  |  |
| MYH13 |  |  | tubulin glycylase 3E |  |  |  |  |  |
| myosin head |  |  | tubulin polyglutamylase complex subunit 1 |  |  |  |  |  |
| myosin light chain |  |  | tubulin tyrosine ligase like 1 |  |  |  |  |  |
| NAD(P)H-hydrate epimerase |  |  | tubulin tyrosine ligase like 13 |  |  |  |  |  |
| NEK1 |  |  | tubulin tyrosine ligase like 2 |  |  |  |  |  |
| NEK3 |  |  | tubulin tyrosine ligase like 6 |  |  |  |  |  |
| NEK4 |  |  | ubiquitin-activating enzyme e1 family protein |  |  |  |  |  |
| nephrocystin-4 |  |  | Ubiquitin-activating enzyme |  |  |  |  |  |
| neurobeachin |  |  | ubiquitin-protein ligase E3C-like |  |  |  |  |  |
| niemann-pick c1 protein |  |  | ZWI |  |  |  |  |  |
| nubp1 |  |  |  |  |  |  |  |  |
| nuclear protein kinase 2 |  |  |  |  |  |  |  |  |
| nucleoside diphosphate kinase |  |  |  |  |  |  |  |  |
| nudC |  |  |  |  |  |  |  |  |
| nudC domain-containing protein 3 |  |  |  |  |  |  |  |  |
| OFD1 |  |  |  |  |  |  |  |  |
| origin recognition complex subunit 1 |  |  |  |  |  |  |  |  |
| OSCP1 |  |  |  |  |  |  |  |  |
| outer dense fiber protein 3 |  |  |  |  |  |  |  |  |
| OXCT1 |  |  |  |  |  |  |  |  |
| Oxysterol-binding protein 1 |  |  |  |  |  |  |  |  |
| PABPN1 |  |  |  |  |  |  |  |  |
| PDE2A |  |  |  |  |  |  |  |  |
| PF20 |  |  |  |  |  |  |  |  |
| pfkb |  |  |  |  |  |  |  |  |
| PFKL |  |  |  |  |  |  |  |  |
| phosphatidylinositol 4-kinase |  |  |  |  |  |  |  |  |
| Phosphoglycerate kinase |  |  |  |  |  |  |  |  |
| Phosphoglycerate mutase |  |  |  |  |  |  |  |  |
| phospholipid-transporting ATPase IM |  |  |  |  |  |  |  |  |
| PI4K2A |  |  |  |  |  |  |  |  |
| PIBF1 |  |  |  |  |  |  |  |  |
| PIH1 domain containing protein |  |  |  |  |  |  |  |  |
| PIK3R4 |  |  |  |  |  |  |  |  |
| PKD2L1 |  |  |  |  |  |  |  |  |
| PLK1 |  |  |  |  |  |  |  |  |
| PLK3 |  |  |  |  |  |  |  |  |
| POC1A |  |  |  |  |  |  |  |  |
| polo-like kinase 1 |  |  |  |  |  |  |  |  |
| Polycystin 2 |  |  |  |  |  |  |  |  |
| PP1 |  |  |  |  |  |  |  |  |
| PP2C |  |  |  |  |  |  |  |  |
| PPP1R7 |  |  |  |  |  |  |  |  |
| PPP2R1B |  |  |  |  |  |  |  |  |
| PPP2R3C |  |  |  |  |  |  |  |  |
| PPP3CB |  |  |  |  |  |  |  |  |
| Protein casc1 |  |  |  |  |  |  |  |  |
| protein IMPACT |  |  |  |  |  |  |  |  |
| Protein-tyrosine phosphatase containing protein |  |  |  |  |  |  |  |  |
| prp4 |  |  |  |  |  |  |  |  |
| PSMD8 |  |  |  |  |  |  |  |  |
| PTPN12 |  |  |  |  |  |  |  |  |
| pyruvate kinase |  |  |  |  |  |  |  |  |
| rab gdp dissociation inhibitor |  |  |  |  |  |  |  |  |
| RAB10 |  |  |  |  |  |  |  |  |
| RAB11a |  |  |  |  |  |  |  |  |
| RAB11B |  |  |  |  |  |  |  |  |
| RAB14 |  |  |  |  |  |  |  |  |
| RAB1A |  |  |  |  |  |  |  |  |
| RAB22A |  |  |  |  |  |  |  |  |
| RAB25 |  |  |  |  |  |  |  |  |
| RAB28 |  |  |  |  |  |  |  |  |
| RAB2B |  |  |  |  |  |  |  |  |
| RAB3 |  |  |  |  |  |  |  |  |
| Rab3D |  |  |  |  |  |  |  |  |
| Rab6 |  |  |  |  |  |  |  |  |
| Rab6B |  |  |  |  |  |  |  |  |
| RAB7a |  |  |  |  |  |  |  |  |
| Rab8 |  |  |  |  |  |  |  |  |
| RABL2A |  |  |  |  |  |  |  |  |
| RANBP2 |  |  |  |  |  |  |  |  |
| ras-related protein |  |  |  |  |  |  |  |  |
| RCC1 |  |  |  |  |  |  |  |  |
| rio1 |  |  |  |  |  |  |  |  |
| rnf13 |  |  |  |  |  |  |  |  |
| RPS6KA2 |  |  |  |  |  |  |  |  |
| RSPH |  |  |  |  |  |  |  |  |
| RSPH1 |  |  |  |  |  |  |  |  |
| RSPH14 |  |  |  |  |  |  |  |  |
| RSPH3 |  |  |  |  |  |  |  |  |
| RSPH9 |  |  |  |  |  |  |  |  |
| ruvb-like 1-like |  |  |  |  |  |  |  |  |
| RuvB-like 2 |  |  |  |  |  |  |  |  |
| saccharopine dehydrogenase |  |  |  |  |  |  |  |  |
| SAPK2 |  |  |  |  |  |  |  |  |
| SEC61A1 |  |  |  |  |  |  |  |  |
| serine threonine-protein phosphatase 5 |  |  |  |  |  |  |  |  |
| serine/threonine protein kinase |  |  |  |  |  |  |  |  |
| Serine/threonine protein kinase 2 |  |  |  |  |  |  |  |  |
| serine/threonine protein phosphatase |  |  |  |  |  |  |  |  |
| Serine/threonine-protein kinase fray2 |  |  |  |  |  |  |  |  |
| SF3A1 |  |  |  |  |  |  |  |  |
| SF3B2 |  |  |  |  |  |  |  |  |
| Sgk2 |  |  |  |  |  |  |  |  |
| SLC9A7 |  |  |  |  |  |  |  |  |
| snare domain containing protein |  |  |  |  |  |  |  |  |
| snf1 |  |  |  |  |  |  |  |  |
| Sodium channel protein type 5 subunit alpha |  |  |  |  |  |  |  |  |
| SPAG17 |  |  |  |  |  |  |  |  |
| SPAG6 |  |  |  |  |  |  |  |  |
| SPATA17 |  |  |  |  |  |  |  |  |
| SPEF2 |  |  |  |  |  |  |  |  |
| STK3 |  |  |  |  |  |  |  |  |
| STK7 |  |  |  |  |  |  |  |  |
| synaptojanin-1 |  |  |  |  |  |  |  |  |
| tankyrase |  |  |  |  |  |  |  |  |
| TAP42 domain containing protein |  |  |  |  |  |  |  |  |
| tbc |  |  |  |  |  |  |  |  |
| tbc1 domain family member 22B |  |  |  |  |  |  |  |  |
| t-complex protein 1 subunit alpha |  |  |  |  |  |  |  |  |
| T-complex protein 1 subunit gamma |  |  |  |  |  |  |  |  |
| Tctex1 |  |  |  |  |  |  |  |  |
| Tectonic-1 |  |  |  |  |  |  |  |  |
| testis-expressed protein 9 |  |  |  |  |  |  |  |  |
| TFIIS |  |  |  |  |  |  |  |  |
| thioredoxin |  |  |  |  |  |  |  |  |
| thioredoxin-2 |  |  |  |  |  |  |  |  |
| TITIN |  |  |  |  |  |  |  |  |
| TMEM216 |  |  |  |  |  |  |  |  |
| TMEM231 |  |  |  |  |  |  |  |  |
| TPR2 |  |  |  |  |  |  |  |  |
| TRAF3-interacting protein 1 |  |  |  |  |  |  |  |  |
| trafficking protein particle complex subunit 3 |  |  |  |  |  |  |  |  |
| transcription factor e2f |  |  |  |  |  |  |  |  |
| transcriptional activator myb-like |  |  |  |  |  |  |  |  |
| translation elongation factor 1-alpha |  |  |  |  |  |  |  |  |
| transportin 1 |  |  |  |  |  |  |  |  |
| TRPM4 |  |  |  |  |  |  |  |  |
| TSSC1 |  |  |  |  |  |  |  |  |
| TTC21b |  |  |  |  |  |  |  |  |
| TTC26 |  |  |  |  |  |  |  |  |
| TTC27 |  |  |  |  |  |  |  |  |
| TTC4 |  |  |  |  |  |  |  |  |
| TTC5 |  |  |  |  |  |  |  |  |
| TTC8 |  |  |  |  |  |  |  |  |
| TTLL1 |  |  |  |  |  |  |  |  |
| TTLL11 |  |  |  |  |  |  |  |  |
| TTLL5 |  |  |  |  |  |  |  |  |
| ttll6 |  |  |  |  |  |  |  |  |
| TTLL7 |  |  |  |  |  |  |  |  |
| TTLL8 |  |  |  |  |  |  |  |  |
| tubby-related protein 2 |  |  |  |  |  |  |  |  |
| tubby-related protein 3 |  |  |  |  |  |  |  |  |
| tubulin epsilon chain |  |  |  |  |  |  |  |  |
| tubulin folding cofactor |  |  |  |  |  |  |  |  |
| tubulin folding cofactor C |  |  |  |  |  |  |  |  |
| tubulin folding cofactor D |  |  |  |  |  |  |  |  |
| tubulin specific chaperone d |  |  |  |  |  |  |  |  |
| tubulin tyrosine ligase like 12 |  |  |  |  |  |  |  |  |
| Ubiquitin |  |  |  |  |  |  |  |  |
| ubiquitin hect domain family protein |  |  |  |  |  |  |  |  |
| Ubiquitin-activating enzyme |  |  |  |  |  |  |  |  |
| ULK3 |  |  |  |  |  |  |  |  |
| UMP-CMP kinase |  |  |  |  |  |  |  |  |
| UNC119 |  |  |  |  |  |  |  |  |
| UPL |  |  |  |  |  |  |  |  |
| Vacuolar sorting protein 35 |  |  |  |  |  |  |  |  |
| voltage-dependent N-type calcium channel subunit alpha-1B |  |  |  |  |  |  |  |  |
| V-type proton ATPase subunit C 1 |  |  |  |  |  |  |  |  |
| V-type proton ATPase subunit d 2 |  |  |  |  |  |  |  |  |
| WD domain-containing protein |  |  |  |  |  |  |  |  |
| WD repeat protein 35 |  |  |  |  |  |  |  |  |
| WD repeat-containing protein 19 |  |  |  |  |  |  |  |  |
| WD repeat-containing protein 60 |  |  |  |  |  |  |  |  |
| wd repeat-containing protein 96 |  |  |  |  |  |  |  |  |
| WD repeat-containing protein WRAP73 |  |  |  |  |  |  |  |  |
| WDR69 |  |  |  |  |  |  |  |  |
| xaa-Pro dipeptidase |  |  |  |  |  |  |  |  |
| YWHAH |  |  |  |  |  |  |  |  |
| zinc finger mynd domain-containing protein 12 |  |  |  |  |  |  |  |  |
| zinc finger protein |  |  |  |  |  |  |  |  |
| zinc mynd domain protein 10 |  |  |  |  |  |  |  |  |
