# Supplementary material for: Identification and bioinformatics analysis of cilia-associated gene families in Euplotes amieti (Ciliophora, Hypotrichia)
Source: Front Microbiol. 2025 May 13;16:1486189. doi: 10.3389/fmicb.2025.1486189 (PMC12106347; doi:10.3389/fmicb.2025.1486189)
Supplement: Supplementary file 2 [file Table_2.docx]

**Table S2 Numbers of gene fragments containing homologous cilia-associated genes in four hypotrich ciliates**

| Gene fragments containing homologous genes | Gene family | *E.amieti* | *E.octocarinatus* | *S.lemnae* | *O.trifallax* |
| --- | --- | --- | --- | --- | --- |
| alpha tubulin | **tubulin** | 3 | 13 | 4 | 10 |
| beta tubulin |  | 5 | 16 | 6 | 25 |
| gamma tubulin |  | 1 | 1 | 1 | 2 |
| delta tubulin |  | 1 | 2 | 1 | / |
| epsilon tubulin |  | 1 | / | 1 | 1 |
| Alpha-tubulin N-acetyltransferase |  | 4 | 4 | 5 | 13 |
| tubulin polyglutamylase |  | 12 | 8 | 7 | 15 |
| Gamma-tubulin complex component |  | 3 | 3 | 4 | 1 |
| Tubulin folding cofactor/specific chaperone |  | 6 | 5 | 4 | 10 |
| Tubulin glycylase 3C/3D/3E |  | / | 1 | / | 4 |
| Tubulin tyrosine ligase like |  | 7 | 3 | 7 | 28 |
| Tubulin-tyrosine ligase family protein |  | 74 | 32 | 54 | 62 |
| Tau |  | / | 1 | 2 | 1 |
| cryptic tubulin |  | / | 1 | 1 | 4 |
| MAP |  | 6 | 7 | 8 | 5 |
| CFAP20 | **CFAP** | / | / | / | / |
| CFAP36 |  | 1 | / | 4 | / |
| CFAP45 |  | 1 | / | 1 | 4 |
| CFAP46 |  | 1 | / | / | / |
| CFAP52 |  | 1 | / | / | 2 |
| CFAP58 |  | / | / | / | 5 |
| CFAP61 |  | 1 | / | / | / |
| CFAP69 |  | 1 | / | / | / |
| CFAP7/ |  | 1 | / | / | / |
| CFAP74 |  | 1 | / | / | / |
| CFAP77 |  | 1 | / | / | / |
| CFAP91 |  | 1 | / | 1 | / |
| CFAP206 |  | / | / | 1 | 2 |
| CFAP221 |  | 1 | / | / | / |
| CFAP251 |  | 1 | / | / | / |
| CFAP299 |  | 1 | / | 1 | / |
| CFAP300 |  | 1 | / | / | / |
| CKAP5 |  | 1 | / | 1 | 2 |
| LRRC3 | **LRRC** | 1 | / | / | / |
| LRRC9 |  | 2 | 1 | 2 | 4 |
| LRRC23 |  | 1 | 3 | 1 | 3 |
| LRRC34 |  | / | / | 1 | 2 |
| LRRC40 |  | 1 | 1 | 1 | 2 |
| LRRC43 |  | / | / | 1 | 2 |
| LRRC46 |  | / | 1 | / | 1 |
| LRRC48 |  | 3 | 2 | 1 | 5 |
| LRRC51 |  | 1 | / | 2 | 1 |
| LRRC56 |  | 1 | 3 | 1 | 3 |
| LRRC61 |  | / | 1 | 1 | 2 |
| LRRC |  | 1 | 1 | 6 | 9 |
| TSP | **TSP** | 1 | 1 | 11 | / |
| dynamin | **dynamin** | 11 | 10 | 3 | 2 |
| UBA1/5 | **UBA** | 1 | / | 1 | / |
| E3 ubiquitin-protein ligase | **E3** | 31 | 43 | 45 | 90 |
| E1 | **E1** | 10 | 7 | 6 | 9 |
| IFT20 | **IFT** | / | 1 | 1 | 2 |
| IFT27 |  | 1 | 1 | 1 | 2 |
| IFT46 |  | 2 | 1 | 1 | 3 |
| ITF52 |  | 1 | 1 | 1 | 4 |
| IFT57 |  | 1 | 1 | 1 | / |
| IFT74 |  | 2 | 2 | 1 | 7 |
| IFT80 |  | 1 | 2 | 1 | 1 |
| IFT81 |  | 2 | 2 | 1 | 2 |
| IFT88 |  | 1 | 3 | 1 | 3 |
| IFT122 |  | 1 | 1 | 1 | 2 |
| IFT140 |  | 1 | / | 1 | 6 |
| IFT172 |  | 1 | 1 | 1 | 2 |
| CLUAP1 |  | / | / | / | 2 |
| Dyneins | **CLUAP1** | 60 | 53 | 44 | 132 |
| Kinesins | **Dyneins** | 85 | 69 | 71 | 85 |
| ARF/ARL | **Kinesins** | 14 | 22 | 19 | 52 |
| RAB | **ARF/L** | 44 | 119 | 73 | 126 |
| GDI | **GDI** | 2 | 1 | 1 | / |
| BBS1 | **BBS** | 1 | 1 | 1 | 2 |
| BBS2 |  | 1 | 1 | 1 | 2 |
| BBS3/ARL6 |  | 1 | 1 | 2 | 1 |
| BBS4 |  | 1 | / | 1 | 1 |
| BBS5 |  | 1 | 1 | 1 | 3 |
| BBS7 |  | 1 | / | 1 | 1 |
| BBS8/TTC8 |  | 1 | / | 1 | 1 |
| BBS9 |  | 1 | 1 | 1 | 1 |
| centrin |  | 2 | / | 5 | 3 |
| CEP19 | **CEP** | 1 | 1 | 4 | / |
| CEP41 |  | 1 | 1 | 2 | 2 |
| CEP43 |  | 3 | 2 | 3 | 7 |
| CEP44 |  | / | 2 | 4 | 4 |
| CEP76 |  | 1 | 1 | 1 | / |
| CEP1/4 |  | 2 | 2 | 3 | 4 |
| CEP120 |  | 1 | 2 | 1 | 2 |
| CEP131 |  | 1 | / | 1 | 1 |
| CEP135 |  | 1 | 3 | 1 | 3 |
| CEP162 |  | 1 | / | 2 | 1 |
| CEP290 |  | 1 | / | 1 | 1 |
| central associated prtoein |  | 14 | 12 | 10 | 8 |
| CCDC13 | **CCDC** | 1 | / | 1 | 3 |
| CCDC22 |  | 1 | 1 | 1 | 2 |
| CCDC25 |  | 1 | 1 | 1 | 2 |
| CCDC39 |  | / | / | / | 2 |
| CCDC40 |  | 1 | 1 | 1 | 3 |
| CCDC42 |  | 1 | 2 | 2 | 2 |
| CCDC63 |  | 1 | 1 | 1 | 2 |
| CCDC65 |  | 1 | 1 | 1 | 3 |
| CCDC93 |  | 1 | / | 1 | 1 |
| CCDC94 |  | / | 1 | 1 | 2 |
| CCDC104 |  | / | / | 1 | / |
| CCDC108/CFAP65 |  | 1 | / | 1 | / |
| CCDC113 |  | 1 | 1 | 1 | 3 |
| CCDC146 |  | 1 | 1 | 1 | / |
| CCDC151 |  | 1 | / | 1 | / |
| CCDC153 |  | / | / | 2 | 3 |
| CCDC |  | 1 | / | / | / |
| CCDC176 |  | 1 | / | / | / |
| basal body associated protein |  | 7 | 8 | 3 | 22 |
| RSP | **RSP** | 12 | 13 | 6 | 59 |
| SPEF | **SPEF** | 3 | 1 | 1 | 5 |
| FAP | **FAP** | 9 | 7 | 3 | 24 |
| TTC1 | **TTC** | 1 | 1 | 1 | 1 |
| TTC4 |  | 1 | 1 | 1 | 1 |
| TTC5 |  | 2 | 1 | 1 | 2 |
| TTC16 |  | / | / | 1 | 2 |
| TTC18 |  | 2 | 2 | 2 | 2 |
| TTC21b |  | 1 | / | 1 | 2 |
| TTC26 |  | 2 | 3 | 1 | 4 |
| TTC27 |  | 1 | 2 | 1 | 2 |
| TTC30a |  | / | / | 1 | / |
| others |  | / | 8 | 10 | 13 |
| ADCY | **ADCY** | 8 | 5 | 1 | 2 |
| CK | **CK** | 41 | 23 | 43 | 66 |
| NEK | **NEK** | 4 | 4 | 12 | 18 |
| STPK | **STRK** | 107 | 22 | 118 | 114 |
| MYH | **MYH** | 25 | 9 | 15 | 32 |
| phosphatase (PPR) | **PPR** | 50 | 17 | 44 | 26 |
| polycystin/PKD | **PKD** | 9 | 13 | 5 | 4 |
| INPP | **INPP** | / | / | 2 | / |
| ATP-binding cassette (ABC) | **ABC** | 5 | 5 | 1 | 4 |
| guanine nucleotide-binding protein(GNA) | **GNA** | 2 | 2 | 1 | 16 |
| TRPM | **TRPM** | 26 | 15 | 3 | 4 |
| arrestin(ARR) | **ARR** | 4 | 5 | / | 9 |
| TULP | **TULP** | 2 | 4 | 2 | 3 |
| LRRC | **LRRC** | 11 | 13 | 16 | 34 |
| IQC | **IQC** | 6 | 5 | 5 | 8 |
| ATG1 | **ATG** | 1 | / | / | / |
| ATG3 |  | 1 | / | 1 | / |
| ATG5 |  | 1 | 1 | 1 | 2 |
| ATG7 |  | / | / | / | 1 |
| ATG8 |  | 5 | 5 | 2 | 8 |
| ATG16 |  | 2 | 2 | 3 | 9 |
| ATG18 |  | 1 | / | / | / |
| ATG |  | / | 1 | 2 | 5 |
| TCTN |  | 1 | 2 | / | 2 |
| B9D1/2 | **B9D** | 4 | 4 | 3 | 11 |
| MKS1 | **MKS** | 1 | 1 | 1 | 3 |
| MKS3/TMEM67 |  | 2 | 2 | 2 | / |
| CC2D2A |  | 1 | / | / | 2 |
| TMEM216 | **TMEM** | 1 | / | / | / |
| TMEM231 |  | 2 | 1 | 1 | 1 |
| NPHP4 |  | 1 | 1 | 1 | 2 |
| AHI1 | **AHI** | 2 | 2 | 1 | 3 |
| ﻿RPGRIP1L | **RPGRIP1L** | 1 | / | 2 | / |
